# Disease‐driven domain generalization for neuroimaging‐based assessment of Alzheimer's disease

**DOI:** 10.1002/hbm.26707

**Published:** 2024-05-26

**Authors:** Diala Lteif, Sandeep Sreerama, Sarah A. Bargal, Bryan A. Plummer, Rhoda Au, Vijaya B. Kolachalama

**Affiliations:** ^1^ Department of Computer Science Boston University Boston Massachusetts USA; ^2^ Department of Medicine Boston University Chobanian & Avedisian School of Medicine Boston Massachusetts USA; ^3^ Department of Computer Science Georgetown University Washington DC USA; ^4^ Department of Anatomy & Neurobiology Boston University Chobanian & Avedisian School of Medicine Boston Massachusetts USA; ^5^ Department of Neurology Boston University Chobanian & Avedisian School of Medicine Boston Massachusetts USA; ^6^ Department of Epidemiology Boston University School of Public Health Boston Massachusetts USA; ^7^ The Framingham Heart Study Boston Massachusetts USA; ^8^ Boston University Alzheimer's Disease Research Center Boston Massachusetts USA; ^9^ Faculty of Computing & Data Sciences Boston University Boston Massachusetts USA

**Keywords:** Alzheimer's disease, cognitive impairment, domain generalization, magnetic resonance imaging

## Abstract

Development of deep learning models to evaluate structural brain changes caused by cognitive impairment in MRI scans holds significant translational value. The efficacy of these models often encounters challenges due to variabilities arising from different data generation protocols, imaging equipment, radiological artifacts, and shifts in demographic distributions. Domain generalization (DG) techniques show promise in addressing these challenges by enabling the model to learn from one or more source domains and apply this knowledge to new, unseen target domains. Here we present a framework that utilizes model interpretability to enhance the generalizability of classification models across various cohorts. We used MRI scans and clinical diagnoses from four independent cohorts: Alzheimer's Disease Neuroimaging Initiative (ADNI, *n* = 1821), the Framingham Heart Study (FHS, *n* = 304), the Australian Imaging Biomarkers & Lifestyle Study of Ageing (AIBL, *n* = 661), and the National Alzheimer's Coordinating Center (NACC, *n* = 4647). With this data, we trained a deep neural network to focus on areas of the brain identified as relevant to the disease for model training. Our approach involved training a classifier to differentiate between structural neurodegeneration in individuals with normal cognition (NC), mild cognitive impairment (MCI), and dementia due to Alzheimer's disease (AD). This was achieved by aligning class‐wise attention with a unified visual saliency prior, which was computed offline for each class using all the training data. Our method not only competes with state‐of‐the‐art approaches but also shows improved correlation with postmortem histology. This alignment with the gold standard evidence is a significant step towards validating the effectiveness of DG frameworks, paving the way for their broader application in the field.

## Graphical abstract

By utilizing model interpretability, our deep learning framework enhances assessment of cognitive impairment from MRI scans, paving the way for improved generalizability in machine learning models.

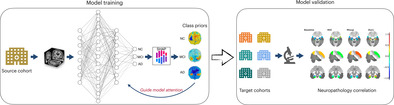

## 1 INTRODUCTION

Dementia due to Alzheimer's disease (AD) is a progressive syndrome leading to loss of brain function that affects memory, thinking, language, judgment and behavior. The approach to dementia diagnosis involves careful consideration of the patient's demographics and symptoms, family, social and medical history, neurologic examination, cognitive, behavioral, and functional assessments along with neuroimaging (Hugo & Ganguli, 2014; McKhann et al., 2011). Magnetic resonance imaging (MRI) is typically recommended to evaluate the structural changes in the patient's brain that correspond to volume loss and atrophy patterns suggestive of AD and rule out other patterns indicative of non‐AD dementias. Computational methods based on advanced machine learning techniques are increasingly considered to automatically process the MRI scans and classify persons with dementia due to AD from those with normal cognition (NC) and mild cognitive impairment (MCI) (Aderghal et al., 2017; Liu et al., 2015; Qiu et al., 2018; Qiu et al., 2020; Qiu et al., 2022). Some of recently reported frameworks have relied on training models using data collected from a single cohort followed by evaluation on independent test cohorts (Qiu et al., 2020). Such model development strategies can establish a proof‐of‐principle, but may lack generalizability because data collected from multiple cohorts contain variabilities stemming from independent scanning protocols, diversity of the study population and other sources. Furthermore, while recent advancements in public data sharing have made data more accessible, there is an increasing necessity to create models that yield findings which are both generalizable and consistent.

Recently, domain generalization (DG) approaches are being considered to train robust deep learning models that account for cohort‐specific variabilities and work well across multiple datasets (Donini et al., 2018; Ghimire et al., 2020; Huang et al., 2020; Koh et al., 2021; Krueger et al., 2021; Li, Pan, et al., 2018; Li, Yang, et al., 2018; Zhang et al., 2020; Zhou et al., 2023). Most methods attempt to mitigate the distributional variance between domain‐specific feature representations. We submit that additional aspects such as orienting the models to focus on disease‐related information while performing model training can be a targeted approach to meet the objective of creating generalizable architectures for disease classification.

### 1.1 Related work

DG frameworks are typically designed to learn a robust signal and a set of patterns possibly from single or multiple source domains with the aim of transferring them to unseen target domains. The expectation is that such frameworks lead to minimal performance degradation on the unseen target environment. In the setting of single‐source DG, the model trained on this source learns robust representations that can generalize to out‐of‐distribution data. Single‐source DG methods can also be applied to a multi‐source setting, as training is done over pooled data across the different source domains (Zhou et al., 2023). Also, multiple source domains can be used for training domain‐invariant feature representations that generalize well to unseen target data.

Most DG methods were originally designed to benchmark natural imaging datasets, with a limited number of frameworks focused on medical imaging data (Ghimire et al., 2020; Koh et al., 2021). A group of methods have been proposed to tackle DG via data manipulation, which could either be data augmentation or generation (Cubuk et al., 2020; Tobin et al., 2017; Volpi et al., 2018; Zhang et al., 2018; Zhou et al., 2020). One of those methods is Mixup (Zhang et al., 2018), a data‐agnostic routine that constructs virtual training examples as convex combinations of pairs of examples and their labels sampled at random from the training distribution. Mixup is designed to regularize the neural network, encouraging it to adopt linear behavior between training examples (Zhang et al., 2018). Another group of methods belong to the use of representation learning to address domain shift, mainly by learning domain‐invariant representations and feature disentanglement (Donini et al., 2018; Ganin et al., 2016; Huang et al., 2020; Krueger et al., 2021; Li, Pan, et al., 2018; Nguyen et al., 2021; Zellinger et al., 2017). Donini and co‐workers proposed a multi‐source algorithm that uses empirical risk minimization (ERM), which became the standard approach to the DG problem (Donini et al., 2018). ERM aims to minimize the training risk across all source domains. Recently, Kreuger and colleagues introduced risk extrapolation (REx) for out‐of‐distribution (OOD) generalization and proposed a penalty on the variance of training risks (V‐REx) (Krueger et al., 2021). They showed that reducing differences in risks with V‐REx can reduce a model's sensitivity to a wide range of extreme distributional shifts. Li et al., on the other hand, proposed using the maximum mean discrepancy (MMD) measure with autoencoders to align distributions across different domains via adversarial training (Li, Pan, et al., 2018). Another work introduced representation self‐challenging (RSC) to force the model to discard dominant features activated on the training data and activate remaining features that correlate with ground‐truth labels (Huang et al., 2020). Further, there exists a line of work that used meta‐learning for DG. One of the proposed meta‐learning methods was MLDG, meta‐learning for domain generalization, which simulates domain shift during training by synthesizing virtual testing domains within each mini‐batch (Li, Yang, et al., 2018).

Our approach represents a distinct advancement from prior research focused on learning domain‐invariant feature representations. It uniquely contributes by employing interpretability techniques to extract disease‐relevant information, which is then used for aligning features effectively. Related prior work used model explanations as means of disentangling domain‐specific information from otherwise relevant features (Zunino et al., 2021). Contrastingly, our method utilizes the feature contributions leading to accurate predictions as a foundation of model‐identified disease biology. This knowledge is then applied to direct the model's focus during training. We concentrate on the single‐source DG setting, which is more practical in clinical environments where the model is trained on a single source domain. The model's ability to generalize is subsequently assessed on external cohorts, which are considered the target domains.

### 1.2 Contributions

Our work falls under the umbrella of medically interpretable machine learning, where we use feature contributions to adjust final predictions by emphasizing disease‐relevant features. Through attention‐based supervision, the model learns to focus on disease‐correlated regions using pre‐computed class‐wise saliency map priors with voxel contributions. The main contributions of this paper are summarized as follows:

- We developed an interpretability‐based computational framework to train deep neural networks that focus on model‐identified disease regions of interest as a means to generalize across multiple cohorts.
- Using MRI scans and clinical data obtained from multiple cohorts, we developed a classifier that distinguishes between persons with NC, MCI and AD.
- We demonstrated that our method competes with state‐of‐the‐art DG methods in the real‐world single‐source setting.
- Finally, we showed that our interpretable findings correlate strongly with postmortem histology, identifying disease presence in brain regions that are known to classically associate with disease.

## 2 METHODS

### 2.1 Study population

We obtained brain MRI scans and corresponding clinical and demographic data on participants from four different cohorts: the Alzheimer's Disease Neuroimaging Initiative (ADNI) $\left(n=1821\right)$ (Petersen et al., 2010), National Alzheimer's Coordinating Center (NACC) $\left(n=4647\right)$ (Beekly et al., 2004), the Australian Imaging Biomarkers & Lifestyle (AIBL) Study of Ageing $\left(n=661\right)$ (Ellis et al., 2009), and the Framingham Heart Study (FHS) (Mahmood et al., 2014; Massaro et al., 2004) $\left(n=304\right)$. There were 3697 cases with normal cognition (NC), 2323 cases with mild cognitive impairment (MCI), and 1413 cases with dementia due to Alzheimer's disease (AD) across all cohorts (Table 1). Statistical analysis of distributional variance, including variance in image quality and imaging equipment, across the four cohorts can be found in Figures S1–S3 of the supplement. Additionally, our study incorporated post‐mortem histological evaluations of 23 participants from the ADNI and FHS cohorts, who deceased within 1 year after their last MRI scan. These assessments comprised pathology grades derived from three distinct stains, which quantified the extent of disease in cortical and subcortical brain structures. In our approach, we adopted a single‐source setting for DG. Here, the training, internal validation, and initial testing of our models were conducted using data from one source cohort. Subsequently, external validation and further testing were carried out on the target cohorts.

**TABLE 1:** Study population.

		Age, years	Education, years	Gender	MMSE	APOE4
Dataset	Group [participants]	Mean [std]	Median [std]	Male count (%)	Median [std]	Positive count (%)
ADNI	NC [*n* = 481]	74.3 ± 6.0	16.3 ± 2.7	235 (48.9%)	29.1 ± 1.1	138 (29.6%)^a^
	MCI [*n* = 971]	72.8 ± 7.7	15.9 ± 2.8	572 (58.9%)	27.6 ± 1.8	438 (47.2%)^a^
	AD [*n* = 369]	74.9 ± 7.8	15.2 ± 3.0	203 (55.0%)	23.2 ± 2.1	229 (64.3%)^a^
	*p*‐value	<.001	<.001	.001	<.001	<.001
NACC	NC [*n* = 2524]	69.8 ± 9.9	15.92 ± 3.0	871 (34.5%)	29.0 ± 1.3	599 (30.0%)^a^
	MCI [*n* = 1175]	74.0 ± 8.7	15.4 ± 3.4	555 (47.2%)	26.8 ± 2.5	322 (38.7%)^a^
	AD [*n* = 948]	75.0 ± 9.1	14.6 ± 3.6	431 (45.5%)	20.5 ± 5.7	346 (52.2%)^a^
	*p*‐value	<.001	<.001	<.001	<.001	<.001
AIBL	NC [*n* = 480]	72.5 ± 6.2	N.A.	203 (42.3%)	28.7 ± 1.2	12 (2.5%)
	MCI [*n* = 102]	74.7 ± 7.1	N.A.	53 (52.0%)	27.1 ± 2.1	12 (11.8%)
	AD [*n* = 79]	73.3 ± 7.8	N.A.	33 (41.8%)	20.4 ± 5.5	14 (17.7%)
	*p*‐value	.006	N.A.	.189	<.001	<.001
FHS	NC [*n* = 212]	73.4 ± 9.6	1.8 ± 0.9^b^	112 (52.8%)	28.1 ± 1.7	42 (20.2%)^a^
	MCI [*n* = 75]	76.2 ± 6.8	1.6 ± 1.0^b^	34 (45.3%)	27.2 ± 2.0	17 (23.6%)^a^
	AD [*n* = 17]	78.8 ± 7.2	1.8 ± 1.0^b^	4 (23.5%)	24.0 ± 2.1	7 (43.8%)^a^
	*p*‐value	.007	.272	.049	<.001	.088

*Note*: MRI scans and corresponding clinical and demographic data were collected across four different cohorts: the Alzheimer's Disease Neuroimaging Initiative (ADNI), the National Alzheimer's Coordinating Center (NACC), the Australian Imaging, Biomarker & Lifestyle Flagship Study of Ageing (AIBL), and the Framingham Heart Study (FHS). The models were trained and tested to differentiate persons who have either normal cognition (NC), mild cognitive impairment (MCI) or dementia due to Alzheimer's disease (AD). Education information on the AIBL dataset was not available.

^a^ Data were not available for some subjects.

^b^ FHS education code: 0 = high school did not graduate, 1 = high school graduate, 2 = some college graduate, 3 = college graduate.

### 2.2 Data selection criterion

To ensure uniformity and control for potential confounding factors, we uniformly applied a set of selection criteria across all cohorts, as detailed in Table 1. These criteria, derived from ADNI's baseline recruitment protocol (Petersen et al., 2010), were crucial in shaping our study's dataset. Our focus was on individuals aged 55 and above, a demographic choice reflective of AD characteristics, including the presence of brain atrophy observable in MRI scans. In our selection process, only subjects with MRI scans conducted within 6 months of their clinically confirmed diagnosis were included, prioritizing the scan closest to the diagnosis date when multiple MRIs were available. We excluded cases involving AD with mixed dementia, non‐AD dementias, a history of severe traumatic brain injury, depression, stroke, brain tumors, or significant systemic illnesses. The MRI scans we analyzed adhered to a strict acquisition protocol, involving a T1‐weighted sequence, 3D acquisition type (irrespective of the acquisition plane), and a field strength of either 1.5 or 3 Tesla. Additionally, most selected cases provided comprehensive demographic information, including gender, age, education level, and details about the scanner manufacturers or brands.

### 2.3 MRI processing and quality assurance pipeline

The MRI scans, downloaded in NIFTI format, underwent a series of preparatory steps to ensure consistency and accuracy before skull‐stripping using the FSL brain extraction tool (BET) (Smith, 2002), and subsequent alignment to the MNI152 template (Fonov et al., 2009). Initially, the scans were oriented to match the MNI template's axis order, utilizing the “fslorient2std” function within FSL. This step was crucial for standardizing the orientation across all scans. Following this, the “robustfov” function estimated the robust field of view, a process that efficiently removed extraneous areas such as the neck and lower head from the scan. This function provided bounding box 3D coordinates of the estimated field of view, crucial for the next processing step. Utilizing these coordinates, the “fslmaths” function cropped the scan to focus on the region of interest, which excluded voxels corresponding to white matter, cerebrospinal fluid, the brain stem, and the cerebellum. This precise cropping was imperative to isolate cerebral regions for in‐depth analysis, ensuring the scans were optimally prepared for the subsequent steps in our study.

Following the initial preparation of the scans, we utilized the FSL brain extraction tool (BET) (Smith, 2002) for skull stripping. The BET function operates with two primary parameters: the fractional intensity threshold (*f*), which ranges between 0 and 1, and the vertical gradient in fractional intensity threshold (*g*), with values spanning from −1 to 1. To assess the quality of the processing, we conducted an inspection of the outputs. This involved generating and analyzing images from randomly selected slices across the axial, sagittal, and coronal planes of each scan. We visually assessed the extracted brain scans and moved the ones with issues such as residual skull fragments or unintended removal of gray matter by BET to a separate group. We then proceeded to reprocess the problematic cases in batches, adjusting the BET parameters to rectify the identified issues. This iterative approach allowed us to fine‐tune the processing settings for improved outcomes. We discovered that setting the *f* value at 0.45 and the *g* value at 0 consistently produced the most accurate and reliable results in skull stripping, significantly enhancing the quality of the processed scans for our subsequent analyses. Finally, we applied intensity normalization and bias field correction to remove any intensity artifacts and increase data homogeneity, then we assessed the quality of the processed MRI scans. Results of the image quality assessment (IQA) can be found in Figure S2 of the supplement. Parcellation was performed on the processed scans of deceased persons from ADNI and FHS $\left(n=23\right)$ with post‐mortem histology who had their last MRI scan taken within 1 year of their death. This was done by applying a nonlinear warp of the Hammersmith Adult brain atlas, segmenting the brain into cortical and subcortical structures, allowing us to study region‐based correlations between model‐derived attention scores and post‐mortem histology.

### 2.4 Computational framework

**FIGURE 1:**
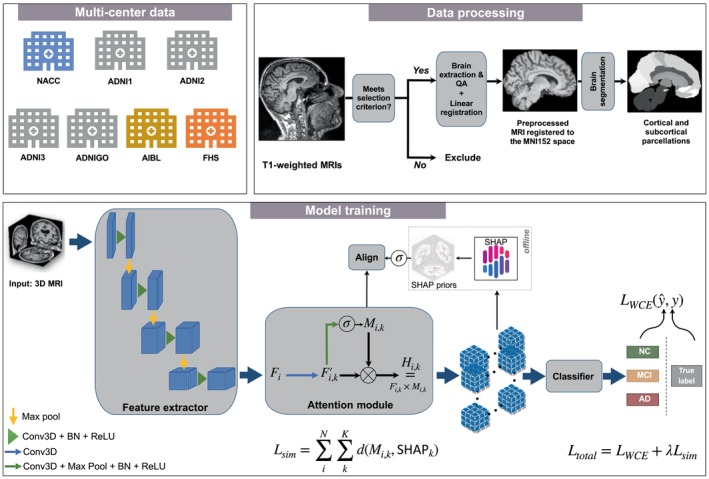
Schematic of the disease‐informed domain generalization framework. MRI scans from various cohorts were processed via an image processing and quality assurance pipeline (see Section 2.3). Segmentation was applied to scans of deceased individuals from the ADNI and FHS cohorts $\left(n=23\right)$ taken within 1 year of their death, with post‐mortem histology available. Our approach takes 3D MRIs as input from the source domain and learns their feature representations in the latent space, and uses an attention module to learn class‐specific saliency maps. These maps are then used to predict a class label (NC, MCI, or AD). We used SHAP offline to generate the averaged saliency maps, which we refer to as disease‐informed prior knowledge, of NC, MCI, and AD classes over all samples of the source domain used for model training.

Our framework is designed for the classification of 3D volumetric brain scans into three distinct cognitive states: Normal Cognition (NC), Mild Cognitive Impairment (MCI), and Alzheimer's Disease (AD). The building blocks of our framework are a feature extractor, a class‐wise attention module, and a classifier network (Figure 1). The training pipeline consists of two stages: the first is training a baseline model for the offline computation of class‐wise priors, and the second is training a new independent model with the supervision of these priors.

#### 2.4.1 Feature extractor

We chose the UNet3D (Çiçek et al., 2016) architecture and started from a pretrained Models Genesis checkpoint on chest CT scans (Zhou et al., 2019; Zhou et al., 2021). Models Genesis are generic pretrained 3D models for 3D medical image analysis. They were trained in a self‐supervised robust manner, and outperformed models trained from scratch (Zhou et al., 2021). To adapt the network to our classification task, we discarded the decoder module and kept the encoder of the UNet3D network as the feature extractor for our framework. Another feature extractor we tried was the transformer‐based Swin UNETR (Hatamizadeh et al., 2022) which employs a state‐of‐the‐art window multi‐head self‐attention mechanism to learn embeddings in the latent space. We utilized pretrained weights yielded by the self‐supervised pretraining of the Swin UNETR encoder on CT scans of the chest, abdomen, and head/neck. The Swin UNETR encoder was pretrained with multiple proxy tasks tailored for medical image representation (Hatamizadeh et al., 2022).

#### 2.4.2 Classifier module

We used a global average pooling (GAP) layer (Lin et al., 2013) followed by a softmax function as the classifier for the three‐way classification of NC, MCI, and AD. Our choice of a GAP layer as opposed to a fully connected layer as the classifier encourages spatial awareness. The latter approach involves inputting a feature map that is pooled over the channel dimension and subsequently flattened into a one‐dimensional vector. In contrast, the former approach processes a stack of 3D feature maps, where the channel dimension K corresponds to the number of classes. This method pools over the spatial dimensions, effectively preserving spatial information for each channel.

#### 2.4.3 Attention supervision

We added an attention module between the feature extractor and the classifier to learn class‐wise attention over the source domain. It takes as input the feature maps $F_{k}$ output by the feature extractor, and passes it through a 3D convolutional layer to get $F_{k}^{\prime }$. The attention maps learned during this process are denoted by $M_{k}\in {\mathbb{R}}^{K\times D\times H\times W}$, where K is the number of classes, and D, H, and W are the depth, height, and width of the attention map, respectively. The final output of the attention module is then the element‐wise multiplication of $F_{k}^{\prime }$ and $M_{k}$. The class‐wise attention maps were later used in the second stage of training for alignment with visual saliency priors computed per class over the training data.

#### 2.4.4 Training

**FIGURE 2:**
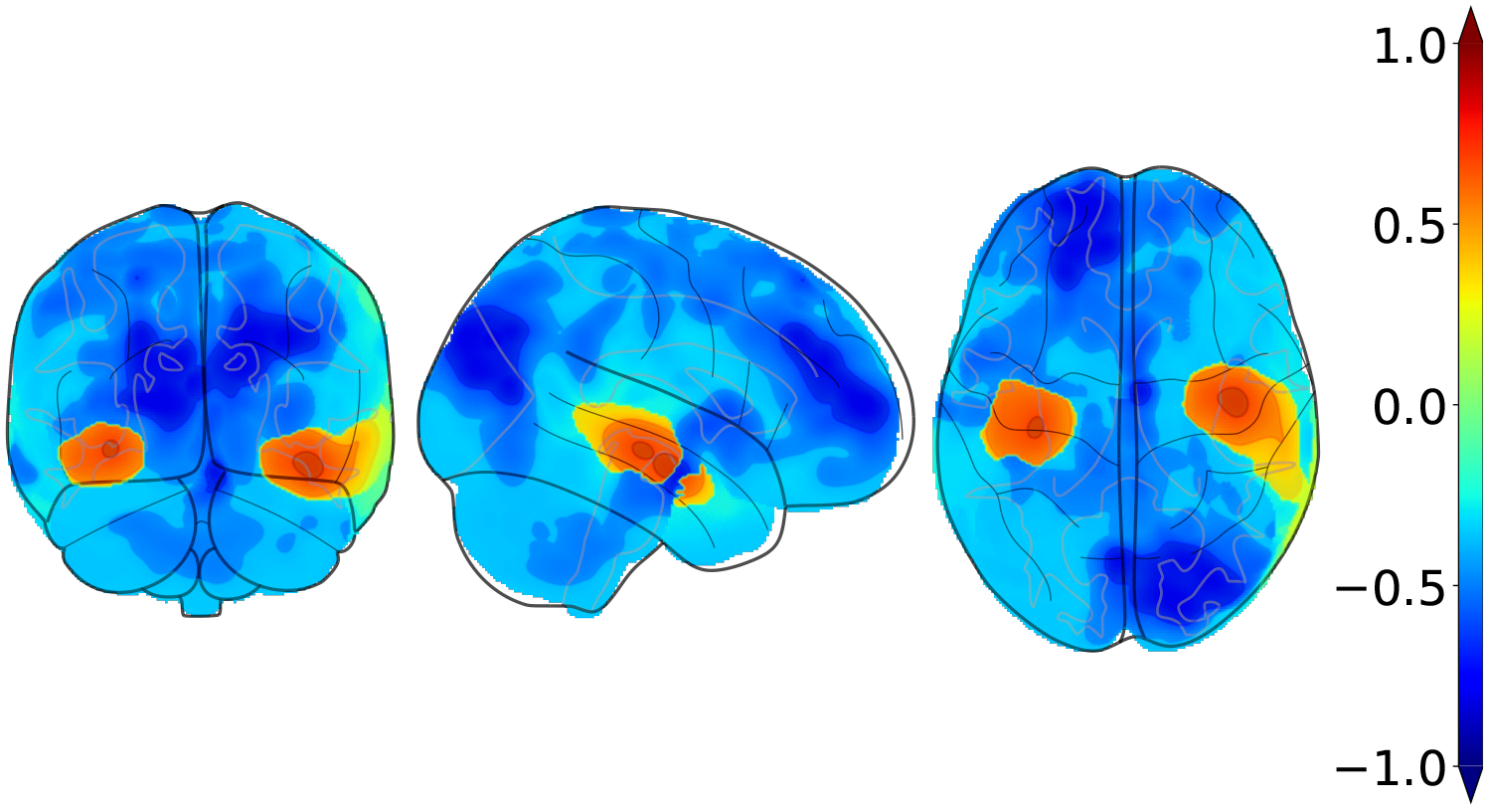
Orthogonal projections of the pre‐computed AD‐specific SHAP priors used in our computational framework. The above projections correspond to the averaged saliency maps with respect to correct predictions of AD over all samples of the source domain. We projected the resulting maps to 2D space onto the coronal, sagittal, and axial axes, respectively.

In the first phase of training, we computed visual saliency maps over correct predictions by a baseline model trained with weighted cross‐entropy over the training data. To achieve this task, we used SHapley Additive exPlanations (SHAP) to compute the feature contributions per class (Lundberg & Lee, 2017). For the purpose of smoothing out sample noise and variance, we used an averaged saliency map over samples of the same class as a representation of class‐wise saliency. Figure 2 shows visualizations of the pre‐computed SHAP priors specific to the AD class. For the purpose of visualization, Shapley values were scaled to the range of $\left[-1,1\right]$, which we chose to correctly represent negative and positive voxel contributions as in the original range. Once the SHAP priors were generated, we ran our explainability‐based strategy to regularize the model through a combined weighted cross entropy (1) and similarity loss (2). We applied augmentation techniques to the training data using the Medical Open Network for AI (MONAI) framework (Cardoso et al., 2022), which included random contrast adjustment, random bias field, random spatial cropping, upsampling, and intensity scaling. We found that intensity scaling to the range $\left[0,1\right]$ worked best for data normalization of structural MRI scans.

(1) $$L_{\mathrm{WCE}}\left(\hat{y},y\right)=-\sum_{i}^{N}w_{y_{i}}.y_{i}\log\left({\hat{y}}_{i}\right),$$

such that N spans the minibatch dimension, and $w_{y_{i}}$ refers to the weight assigned to all samples belonging to the ground‐truth class $y_{i}$. Class weights are computed by taking the inverse of the total count of samples belonging to each class, so that underrepresented classes have a higher weight.

After having the SHAP maps generated offline per class, we used a similarity loss defined in (2) to minimize the distance between each sample's extracted feature maps and the retrieved SHAP prior with respect to the same class as the ground truth label of that sample.

(2) $$L_{\mathrm{sim}}=\sum_{i}^{N}\sum_{k}^{K}d\left(M_{i,k},{\text{SHAP}}_{k}\right),$$

with d being the distance metric of choice, which, in our case, is the L2 norm. We used the L2 norm loss to increase the semantic consistency between the attention maps $M_{i,k}$ and SHAP priors SHAP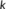 corresponding to class $k\in \left[1,K\right]$, thereby encouraging the model to focus its attention on disease‐relevant regions that the pre‐computed priors highlighted in the brain.

The final loss is then:

(3) $$L=L_{\mathrm{WCE}}+\lambda L_{\mathrm{sim}},$$

where λ is a hyper‐parameter that can be optimized.

### 2.5 Neuropathological validation

To validate model predictions with gold standard biological evidence, we correlated deep feature contributions with region‐specific neuropathological scores obtained from autopsy on persons who had their last MRI within a year of their demise. We quantified regional disease presence based on the degree of amyloid β deposits, neurofibrillary tangles (NFT), and neuritic plaques (NP) on histology. These three pathologies are hallmarks of AD that increase in density and/or spread through the brain as the disease progresses, and they are associated with tissue/cellular damage and death (McKhann et al., 1984).

We obtained 23 participants from ADNI $\left(n=13\right)$ and FHS $\left(n=10\right)$ who had MRI scans taken within 1 year of death with available regional semi‐quantitative histopathology scores. Presence and density of amyloid β deposits, neurofibrillary tangles, and neuritic plaques were assessed in the entorhinal, hippocampal, frontal, temporal, parietal, and occipital cortices. The regions were proposed based on the NIA‐AA protocol for standardized neuropathological assessment of AD. Severity of the assessment was categorized into four score categories: 0 (None), 1 (Mild), 2 (Moderate), and 3 (Severe) (Hyman et al., 2012). We used the trained models to run inference on those cases and saved their corresponding class‐wise attention maps for computation of region‐level scores. Since postmortem histology grades assess for the presence of disease in the respective brain regions, we used the AD‐specific attention map to compute region‐level attention scores as model evidence for the prediction of AD. Using the MNI‐152 template, we obtained a brain parcellation for each of the MRIs and aggregated voxel attention values per region, normalized by regional volume. Once model scores were computed, we ran the Spearman's rank correlation coefficient test with pathology grades of amyloid β, neurofibrillary tangles, and neuritic plaques in the various pre‐identified brain regions. Following (Rothman, 1990; Saville, 1990), the resulting *p*‐values were not adjusted for multiple comparisons.

## 3 EXPERIMENTAL SETUP

We considered the NACC dataset as the source domain for training, validation and internal testing, and used ADNI, AIBL, and FHS as the target domains for external testing. All experiments were run with *k*‐fold cross validation over the source domain with k=5, and the average metrics over the five runs with their standard deviation were reported. Since the source domain we have access to suffers from class imbalance, wherein MCI and AD cases are significantly less than NC cases, we used stratified *k*‐fold cross validation to ensure the target classes follow the same ratio in each fold as in the full dataset. We used a split ratio of 3:1:1, where 60% of the data were used for model training, 20% were used for internal validation, and the rest for internal testing. We trained our models for 60 epochs with 200 steps, that is, weight updates, per epoch. We also compared against two state‐of‐the‐art methods in the single‐source DG setting: RSC (Huang et al., 2020) and Mixup (Zhang et al., 2018). After hyper‐parameter tuning, we chose a $\lambda =5\times 10^{-5}$ for our training strategy and an α=0.2 for the Mixup method. Due to large size of the input image, that is, $\left(182\times 218\times 182\right)$ per MRI, we could only fit a batch size of 2 into GPU memory (48 GB) and had to resort to gradient accumulation over 8 steps to simulate a final batch size of 16, since the small batch size rendered weighted random sampling ineffective for mitigating class imbalance. We also modified the state‐of‐the‐art DG methods to use weighted cross‐entropy across all experiments, as their implementation was not designed to deal with heavy class imbalance.

### 3.1 Performance metrics

Along with model accuracy, we reported the macro F1‐score averaged over five folds as it better represents a balanced score between precision and recall through their harmonic mean. The macro F1‐score in multi‐class classification is the average of F1‐scores over all classes (4). A higher macro F1 score represents lower false positives, that is, recall, and false negatives, that is, precision.

(4) $$\text{Macro}\;F_{1}=\sum_{k}^{K}\frac{2\times {\text{Precision}}_{k}\times {\text{Recall}}_{k}}{{\text{Precision}}_{k}+{\text{Recall}}_{k}}$$

such that,

(5) $$\text{Precisio}n_{k}=\frac{M_{\mathrm{kk}}}{\sum_{i}M_{\mathrm{ik}}}$$

(6) $$\text{Recal}l_{k}=\frac{M_{\mathrm{kk}}}{\sum_{i}M_{\mathrm{ki}}}.$$

We also reported Matthew's Correlation Coefficient (MCC), using Scikit‐Learn's (Kramer, 2016) formula for multi‐class classification (7). An advantage of having MCC as a single‐value classification metric is that it is perfectly symmetric, unlike precision and recall that can be affected by swapping positive and negative classes. In addition, it quantifies how well the model is doing at predicting each class, regardless of class imbalance.

(7) $$\mathrm{MCC}=\frac{c\times s-\sum_{k}^{K}p_{k}\times t_{k}}{\sqrt{\left(s^{2}-\sum_{k}^{K}p_{k}^{2}\right)\times \left(s^{2}-\sum_{k}^{K}t_{k}^{2}\right)}}$$

such that,

(8) $$t_{k}=\sum_{i}^{K}M_{\mathrm{ik}}$$

(9) $$p_{k}=\sum_{i}^{K}M_{\mathrm{ki}}$$

(10) $$c=\sum_{k}^{K}M_{\mathrm{kk}}$$

(11) $$s=\sum_{i}^{K}\sum_{j}^{K}M_{\mathrm{ij}},$$

where M refers to the confusion matrix, K the total number of classes, $t_{k}$ the number of times class k truly occurred, $p_{k}$ the number of times class k was predicted, c the total number of samples correctly predicted, and s the total number of samples.

### 3.2 Computing infrastructure

We used PyTorch (v1.13.1) and a NVIDIA A6000 graphics card with 48 GB memory on a GPU workstation to implement the model. The training speed was about 2.25 s/iteration, and it took less than 24 h to reach convergence with a batch size of 16 after gradient accumulation. The inference speed was <0.2 s per MRI.

### 3.3 Data and code availability

All the MRI scans and corresponding clinical and demographic data can be downloaded freely from ADNI, NACC and AIBL websites. FHS data is available upon request and subject to institutional approval. Python scripts and manuals are available on GitHub.^1^

## 4 RESULTS

We compared the results of our computational framework against state‐of‐the art DG methods for the single‐source setting in Table 2. We used a vanilla UNet3D model trained without DG on the NACC cohort as the baseline on which we ran three different experiments: one trained from scratch and not using attention (Row 1), another also trained from scratch but with our attention module (Row 2), and the third trained starting from the pretrained Models Genesis (Zhou et al., 2019; Zhou et al., 2021) checkpoint with our attention module (Row 3). First, the two methods we compared against, RSC (Huang et al., 2020) and Mixup (Zhang et al., 2018), did not show improvement over the baseline. In fact, performance was deteriorated going from Row 3 to 4 by 10.8% in terms of target mean accuracy, 0.07 $\left(7\%\right)$ in terms of target mean macro F1‐score, and 0.08 $\left(4\%\right)$ in terms of target mean MCC. The same pattern of performance degradation was observed going from Rows 3 to 5, with a 4.6% lower target mean accuracy, a 0.03 $\left(3\%\right)$ lower target mean macro F1‐score, and 0.04 $\left(2\%\right)$ lower target mean MCC. These findings suggest that while these methods have shown enhanced performance and resilience against distributional changes in natural and synthetic imaging benchmarks, their effectiveness may not extend to real‐world clinical scenarios, specifically in the context of volumetric structural brain MRIs. On the other hand, training using our method improved performance, outperforming RSC, Mixup, and the baseline across the reported target mean metrics. We showed a 2.8% improvement over the baseline (Row 3 vs. Row 7) in terms of target mean accuracy. In fact, our method was able to achieve a 73.4% accuracy on the target cohort AIBL, a 7.3% improvement over the baseline (Row 7 vs. Row 3). This improvement is also reflected in the MCC value which increased by 0.07 $\left(3\%\right)$ from Rows 3 to 7. Receiver operating characteristic (ROC) and precision–recall (PR) curves supporting our findings were included in the supplement in Figures S8 and S9, respectively.

**TABLE 2:** Model performance on the classification of NC, MCI, and AD.

Method	Class‐wise attention	Pretrained		Source	ADNI	AIBL	FHS	Target mean
Baseline (Zhou et al., 2019)	✘	✘	Accuracy (%)	52.5 ± 5.4	38.9 ± 2.7	54.1 ± 11.5	38.6 ± 10.0	43.9 ± 6.9
Baseline (Zhou et al., 2019)	✓	✘		52.7 ± 2.5	42.9 ± 0.6	52.6 ± 4.8	42.7 ± 4.5	46.1 ± 2.5
Baseline (Zhou et al., 2019)	✓	✓		64.3 ± 4.0	42.7 ± 1.4	66.1 ± 3.7	48.1 ± 7.2	52.3 ± 2.9
RSC (Huang et al., 2020)	✓	✓		55.5 ± 3.4	43.8 ± 3.8	44.8 ± 9.0	35.9 ± 9.2	41.5 ± 3.0
Mixup (Zhang et al., 2018)	✓	✓		62.8 ± 1.5	43.5 ± 2.0	65.2 ± 3.6	34.3 ± 2.8	47.7 ± 2.1
Ours	✓	✘		51.6 ± 2.3	**44.0 ± 0.4**	47.3 ± 3.3	37.1 ± 3.6	42.8 ± 1.6
Ours	✓	✓		**66.5 ± 1.3**	42.9 ± 1.5	**73.4 ± 2.4**	**49.1 ± 6.5**	**55.1 ± 2.9**
Baseline (Zhou et al., 2019)	✘	✘	Macro F1 Score	0.50 ± 0.04	0.39 ± 0.03	0.44 ± 0.06	0.33 ± 0.07	0.39 ± 0.05
Baseline (Zhou et al., 2019)	✓	✘		0.50 ± 0.02	0.44 ± 0.01	0.45 ± 0.02	0.37 ± 0.03	0.42 ± 0.01
Baseline (Zhou et al., 2019)	✓	✓		0.58 ± 0.02	0.44 ± 0.02	0.54 ± 0.02	0.40 ± 0.05	0.46 ± 0.02
RSC (Huang et al., 2020)	✓	✓		0.52 ± 0.01	0.44 ± 0.03	0.42 ± 0.02	0.32 ± 0.07	0.39 ± 0.02
Mixup (Zhang et al., 2018)	✓	✓		0.58 ± 0.01	0.44 ± 0.02	0.54 ± 0.03	0.30 ± 0.02	0.43 ± 0.02
Ours	✓	✘		0.50 ± 0.02	**0.45 ± 0.00**	0.42 ± 0.02	0.34 ± 0.03	0.40 ± 0.01
Ours	✓	✓		**0.60 ± 0.02**	0.44 ± 0.02	**0.58 ± 0.02**	**0.41 ± 0.04**	**0.48 ± 0.02**
Baseline (Zhou et al., 2019)	✘	✘	MCC	0.27 ± 0.04	0.13 ± 0.03	0.21 ± 0.06	0.11 ± 0.06	0.15 ± 0.05
Baseline (Zhou et al., 2019)	✓	✘		0.26 ± 0.04	0.18 ± 0.02	0.21 ± 0.03	0.13 ± 0.03	0.17 ± 0.02
Baseline (Zhou et al., 2019)	✓	✓		0.40 ± 0.04	**0.21 ± 0.03**	0.34 ± 0.02	0.19 ± 0.37	0.25 ± 0.02
RSC (Huang et al., 2020)	✓	✓		0.31 ± 0.02	0.18 ± 0.02	0.23 ± 0.01	0.10 ± 0.03	0.17 ± 0.01
Mixup (Zhang et al., 2018)	✓	✓		0.39 ± 0.02	0.19 ± 0.01	0.33 ± 0.03	0.11 ± 0.02	0.21 ± 0.02
Ours	✓	✘		0.26 ± 0.04	0.18 ± 0.01	0.18 ± 0.02	0.11 ± 0.03	0.16 ± 0.02
Ours	✓	✓		**0.42 ± 0.02**	**0.21 ± 0.03**	**0.40 ± 0.02**	**0.20 ± 0.03**	**0.27 ± 0.03**

*Note*: We trained our model on the NACC cohort and used the ADNI, AIBL, and FHS cohorts as target domains. We reported accuracy on the test split of NACC, and on the entirety of the target datasets. Performance metrics including accuracy, macro F1‐score and MCC are reported on each case. Note that model training was done via 5‐fold cross validation on the NACC dataset, and testing was done on each of the models. Results are reported as mean ± standard deviation. The bold font is used to report the best model performance in each column.

**FIGURE 3:**
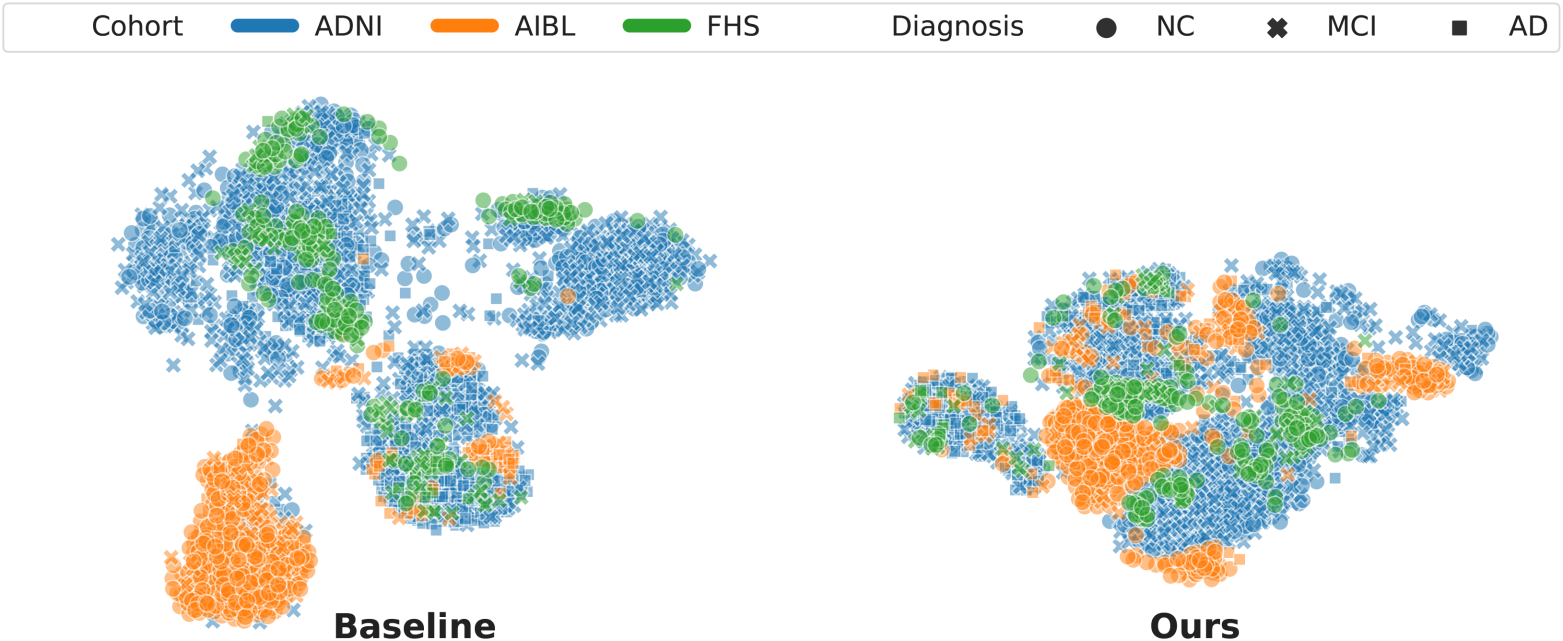
Visualization of MRI embeddings in the latent space. We generated MRI embeddings at the attention module level from two UNet3D models trained on the NACC cohort without domain generalization (Baseline, Row 3 in Table 2) and with our proposed DG framework (Ours, Row 7 in Table 2), and visualized them in a 2D space using t‐SNE. For both models, data from the target cohorts (ADNI $\left(n=1,821\right)$, AIBL $\left(n=661\right)$ and FHS $\left(n=304\right)$) were used. The data points were color‐coded by diagnosis label and marked by cohort.

The above quantitative results were reflected in Figure 3, where we used the t‐distributed stochastic neighbor embedding (t‐SNE) algorithm (Van der Maaten & Hinton, 2008) to visualize MRI embeddings learnt by the baseline model trained without DG (Row 3 in Table 2) and the model trained with our computational framework (Row 7 in Table 2). While the baseline t‐SNE plot shows the MRI embeddings learned by the baseline model clustered by cohort, ours shows that our approach to aligning model attention with SHAP priors reduces cohort‐specific clustering. In particular, the improvement in accuracy over the baseline on the AIBL cohort shows in the dispersion of MRI embeddings belonging to AIBL (orange) across the tSNE plot on the right (Ours) as opposed to a clear cluster highlighted in the plot to the left (Baseline). These results indicate that even though the SHAP priors used in training were derived only from the source domain, they effectively reduced the distributional variance across source and target domains. Moreover, we explored the effect of demographic variance on model performance and included a detailed comparison of our model (Row 7 in Table 2) against the baseline (Zhou et al., 2019) (Row 1 in Table 2) in the supplement (Figures S4–S7). Our model exhibited an overall improvement in performance over the baseline across different distributions of demographic groups.

For comparison with state‐of‐the‐art, results of additional experiments were reported in Table 3 on the ternary classification task of NC, MCI, and AD with the transformer‐based Swin UNETR (Hatamizadeh et al., 2022) encoder as the feature extractor. The model was trained with different classifiers, with and without the class‐wise attention module described in Section 2.4. Table 3 shows a similar performance to the results with the UNet3D encoder in Table 2. Adding the class‐wise attention module exhibited the same trend in performance as reported in Table 2 with the UNet3D (Çiçek et al., 2016) feature extractor. Remarkably, the results in Table 3 show that using a feature extractor with inherent, state‐of‐the‐art self‐attention did not provide an advantage over using class‐wise attention supervision designed to focus on disease biology.

**TABLE 3:** Performance results of training without domain generalization (DG) using the Swin UNETR (Hatamizadeh et al., 2022) encoder as the feature extractor and different classifiers listed below.

Classifier module	Class‐wise attention	Pretrained		Source	ADNI	AIBL	FHS	Target mean
Conv3D	✘	✓	Accuracy (%)	57.1 ± 5.4	35.2 ± 4.8	41.9 ± 9.2	56.6 ± 9.2	44.6 ± 6.4
Conv3D (*N* = 3)	✘	✓		57.2 ± 4.4	44.3 ± 4.4	41.0 ± 2.7	54.9 ± 7.2	46.7 ± 3.2
Conv3D	✓	✓		57.7 ± 1.1	40.7 ± 2.4	39.1 ± 2.0	58.2 ± 2.3	46.0 ± 1.4
GAP	✓	✓		59.8 ± 2.7	41.8 ± 2.9	44.6 ± 5.1	58.4 ± 2.7	48.3 ± 3.1
Conv3D	✘	✓	Macro F1‐score	0.48 ± 0.05	0.34 ± 0.05	0.32 ± 0.05	0.38 ± 0.07	0.34 ± 0.05
Conv3D (*N* = 3)	✘	✓		0.55 ± 0.03	0.43 ± 0.03	0.36 ± 0.01	0.45 ± 0.05	0.41 ± 0.03
Conv3D	✓	✓		0.54 ± 0.01	0.40 ± 0.02	0.34 ± 0.02	0.46 ± 0.02	0.40 ± 0.02
GAP	✓	✓		0.55 ± 0.01	0.42 ± 0.03	0.37 ± 0.04	0.44 ± 0.03	0.41 ± 0.03
Conv3D	✘	✓	MCC	0.30 ± 0.04	0.15 ± 0.03	0.16 ± 0.04	0.17 ± 0.05	0.16 ± 0.03
Conv3D (*N* = 3)	✘	✓		0.35 ± 0.04	0.16 ± 0.02	0.17 ± 0.01	0.21 ± 0.05	0.18 ± 0.02
Conv3D	✓	✓		0.33 ± 0.01	0.13 ± 0.01	0.15 ± 0.01	0.20 ± 0.03	0.16 ± 0.02
GAP	✓	✓		0.35 ± 0.02	0.15 ± 0.02	0.18 ± 0.04	0.18 ± 0.05	0.17 ± 0.03

*Note*: The weights of the feature extractor were loaded from a pretrained checkpoint and fine‐tuned while training on the classification of NC, MCI, and AD. The feature extractor has a window multi‐head self‐attention mechanism built in, and we ran training with and without the class‐wise attention module before the classifier.

**FIGURE 4:**
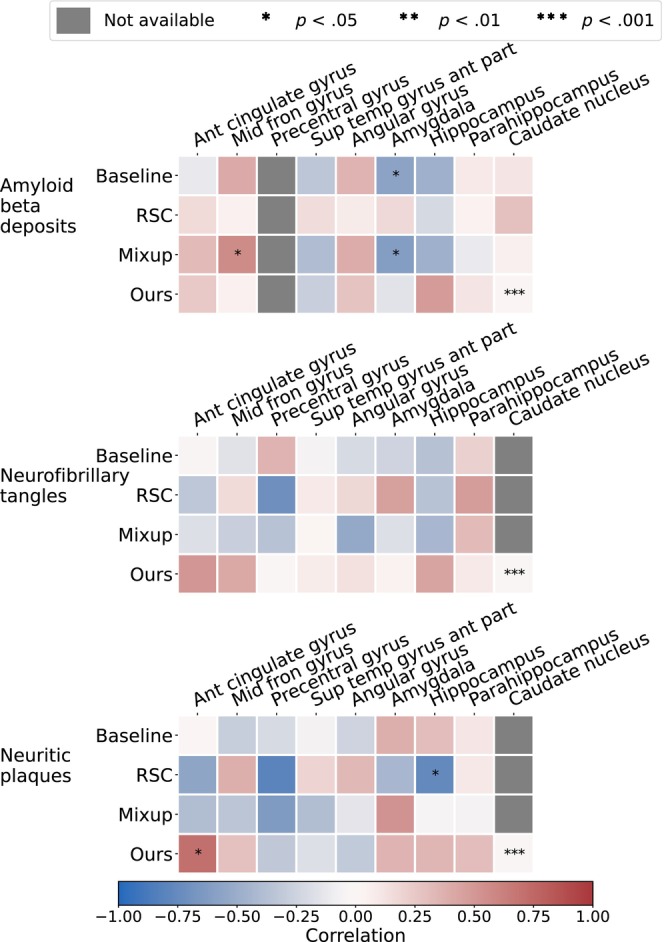
Correlation of model‐generated attention scores with post‐mortem histology. Pathology grades of amyloid β, neurofibrillary tangles and neuritic plaques in various brain regions on deceased ADNI and FHS participants were obtained $\left(n=23\right)$. We compared model‐identified importance in these brain regions with the degree of pathology severity, and compared them against predictions obtained using other well‐known domain generalization methods.

**FIGURE 5:**
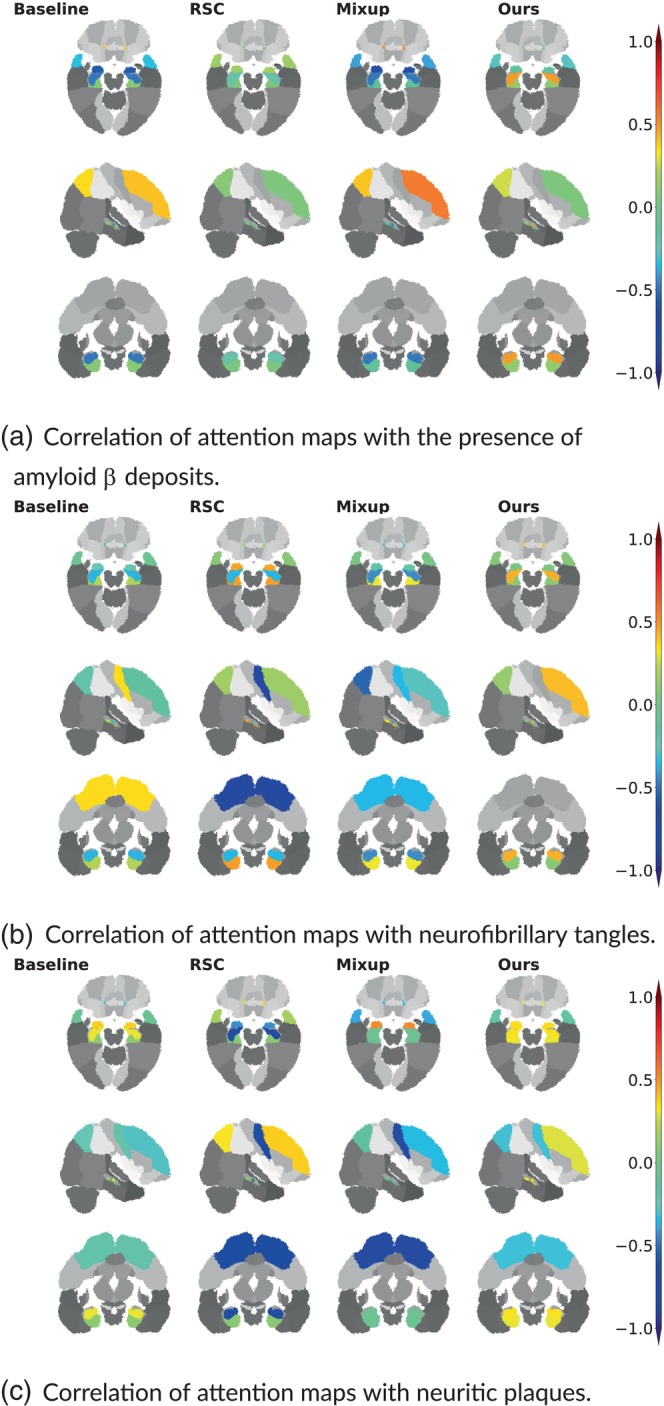
Visualization of correlations between model attention scores and post‐mortem histology. We obtained region‐specific pathology grades of amyloid β, neurofibrillary tangles and neuritic plaques on deceased ADNI and FHS participants $\left(n=23\right)$. The pathology grades reflect the severity of the assessment which was categorized into four score categories: 0 (none), 1 (mild), 2 (moderate), 3 (severe). We obtained attention scores for each case from the model attention maps specific to AD, aggregated on a region level. We then computed Spearman's rank correlation coefficient between the model‐derived attention scores and the region‐specific pathology grades and projected them on the corresponding brain regions for visualization.

We further validated our method with gold standard evidence of disease pathology and compared it against the other methods, reporting the results in the form of a correlation heat map in Figure 4. We showed that not only did our method correlate more strongly with postmortem histology scores than other methods, but also, our results were more consistent across the three stains. Correlation of our method with pathology in the amygdala, hippocampus, parahippocampal and ambient gyri was positive for amyloid β, neurofibrillary tangles, and neuritic plaques. We then projected the computed correlation values onto their corresponding brain regions and displayed the projections (Figure 5). Figure 5a shows an improved correlation for our method with pathology grades of amyloid β in the hippocampal region and the middle frontal gyrus of the frontal lobe. Correlation in these brain regions is also consistent with pathology grades of neurofibrillary tangles and neuritic plaques (Figure 5b,c). As for the other evaluated methods, shown in the first three columns of each subfigure, the correlations were lower with pathology grades in the hippocampus of amyloid β, neurofibrillary tangles, and neuritic plaques, except for the baseline method in Figure 5c that had a positive—although lower than ours—correlation. In addition, our method showed the highest correlation in the parahippocampal and ambient gyri with pathology grades of neuritic plaques in Figure 5c. Our method demonstrated high correlations with specific brain regions, notably the hippocampal and parahippocampal areas, which were visually represented in the pre‐computed AD‐specific SHAP priors (Figure 2). These regions contributed positively to model predictions of AD, indicating the effectiveness of our technique in aligning model attention with established knowledge regarding disease indicators. Such observations indicating improved model correlation with regions that are well‐known to be implicated with disease grounded our model predictions with biological evidence.

## 5 DISCUSSION

This work presents a computational framework for DG that adds disease‐driven interpretability to deep learning models for AD prediction on volumetric MRI scans. While most of the existing methods focus on achieving high model performance on unseen data, they do not directly account for the underlying disease biology during model development. We achieved this goal by refining the model's attention to focus on brain regions that are most associated with disease based on pre‐computed feature contributions. In such fashion, our method distinguishes itself by incorporating disease‐driven interpretability into the training process. The added interpretability can provide a better understanding of the underlying disease mechanisms and aid in the clinical decision‐making process. We compared the performance of our method with previously published DG frameworks, and showed that our approach shows competitive performance while incorporating disease relevance into the model training process. We confirmed the degree to which our attention‐based supervision strategy ultimately reflected disease biology by comparing model attention in predefined brain regions with postmortem neuropathology scores. Overall, our approach to creating a generalizable framework complements other published work in the literature.

We observed that our model achieved consistent, favorable performance on the test cohorts relative to other DG frameworks. While extensive testing is required to confirm any modeling framework's superiority in accurate prediction of disease, it is worth noting that model performance based on accuracy alone without downstream evidence of correlation with a reference standard may not be appealing in the context of medical machine learning. As such, classifying persons with NC from those who have MCI or AD solely on MRIs is a clinically challenging task, and often not part of routine clinical neurology work‐up. Neurologists use a spectrum of patient data along with MRIs to assess a person's cognitive status. Nevertheless, our proposed framework has utility in the objective interpretation of brain MRIs, and broadly in the quantification of findings indicative of disease. Besides minimizing subjectivity, it also potentially fills gaps in healthcare settings where there is a lack of neuroradiology expertise.

Our study has a few limitations. Due to memory limitations, we resorted to offline computation of the saliency maps based on correct predictions by the trained baseline model. We also acknowledge that SHAP prior computation is solely dependent on the baseline model used, that is, the quality of prior knowledge and correctness of feature contributions extracted from the model are directly correlated with model performance. Also, it is possible that the offline computation and aggregation of class‐specific SHAP maps may have reduced instance‐to‐instance variability and minimized radiologic artifacts, thereby facilitating model attention on disease pathology. In addition, it is possible that the model was able to capture the fine‐grained nature of disease markers due to our choice of the voxel‐wise L2 distance metric. We utilized this loss function to increase the semantic similarity between model attention and prior maps at the voxel level.

In conclusion, our work contributes to the growing field of interpretable deep learning in medical imaging, paving the way for more accurate and personalized diagnoses of cognitive disorders. By highlighting the specific brain regions that contribute most significantly to disease, our approach can provide valuable insight into disease mechanisms and aid in developing targeted interventions. Furthermore, the disease‐driven interpretability of our framework can help build trust and understanding between clinicians and patients, which is crucial for effective healthcare delivery.

## AUTHOR CONTRIBUTIONS

DL, SAB, BAP, and VBK: study conception and design. DL and SS: data collection and processing. DL: implementation. DL and SS: analysis. DL, SS, SAB, BAP, RA, and VBK: data interpretation and manuscript write‐up. VBK: study direction. All authors reviewed the results and approved the final version of the manuscript.

## CONFLICT OF INTEREST STATEMENT

V.B.K. is on the scientific advisory board for Altoida Inc., and serves as a consultant to AstraZeneca. R.A. is a scientific advisor to Signant Health and NovoNordisk. She also serves as a consultant to Davos Alzheimer's Collaborative. The remaining authors declare no competing interests.

## Supporting information


**Data S1.** Supplementary information.

## Data Availability

The data that support the findings of this study are available from the corresponding author upon reasonable request.

## Footnotes

^1^

https://github.com/vkola-lab/hbm2024.
