# Supplementary material for: Disease‐driven domain generalization for neuroimaging‐based assessment of Alzheimer's disease
Source: Hum Brain Mapp. 2024 May 26;45(8):e26707. doi: 10.1002/hbm.26707 (PMC11128757; doi:10.1002/hbm.26707)
Supplement: Supplementary file 1 — Data S1. Supplementary information. [file HBM-45-e26707-s001.pdf]

SUPPLEMENT

Disease-driven domain generalization for the neuroimaging-based assessment of Alzheimer's disease

Diala Lteif[1,2] | Sandeep Sreerama[2] | Sarah A. Bargal[3] | Bryan A. Plummer[1] | Rhoda Au[2,4,5] | Vijaya B. Kolachalama[1,2,5,6]

A | STUDY POPULATION

To elucidate the demographic distributions within our study's four cohorts, we utilized box plots in Fig. S1 to display patient demographics: age, education, gender, and the presence of the ApoE4 gene, known to be a genetic risk factor for AD. These results highlight statistical differences among groups with NC, MCI and AD within the four cohorts. We employed the non-parametric Mann-Whitney U Test, applying the Bonferroni correction for multiple comparisons, to derive adjusted p-values indicating significant differences.

The box plots illustrate variations in demographic distributions among different diagnostic groups and cohorts. Notably, the NACC cohort exhibits a distinct presence of outlier values in age and education not observed in other cohorts. Statistical analysis reveals significant differences in demographic characteristics across different diagnoses and cohorts, particularly for age distributions among MCI cases between the NACC and ADNI cohorts and between ADNI and FHS, as well as for NC cases between NACC and the AIBL and FHS cohorts and between ADNI and AIBL. Furthermore, comparisons of education distributions reveal remarkably significant disparities between NACC and ADNI for the NC and MCI groups. Differences in the mean percentage of males and the percentage of ApoE4 positive individuals are also detailed in plots S1c and S1d, respectively, underscoring the variance in demographic and genetic factors across cohorts.

Furthermore, we examined any variabilities in the intensity values, MRI scan image quality, and imaging equipment across the four cohorts after they have been processed. Regarding differences in the image quality, we note that our processing pipeline included intensity normalization and bias field correction, following Qiu *et al.* [1] who showed the effectiveness of their MRI harmonization pipeline in ruling out any intensity artifacts and scanner-, site-, or cohort-specific biases that could be learned by a predictive model [1]. More details on the MRI processing and quality assurance pipeline can be found in Section 2.3 of the manuscript. We further evaluated the processed scans from the four cohorts with image quality assessment (IQA). Fig. S2 shows the distribution plots across the four cohorts of two quality metrics we used for assessment. The first is Signal-to-Noise Ratio (SNR), a metric that reflects instrument-related variations such as the magnetic field of strength. For brain MRIs, it is defined as the quotient of the mean signal intensity measured within the gray matter divided by the standard deviation of the values outside the brain [2]. The second is the Entropy Focus Criterion (EFC) defined as Shannon's entropy of voxel intensities normalized to the maximum possibly entropy of the image [3]. EFC is used as a measurement of the blurring and ghosting artifacts induced by head motion. P-values output by the Mann-Whitney U-test were corrected for multiple comparisons using the Bonferroni method for both metrics. The results reflected statistically significant variations in both SNR and EFC distributions between all cohorts, showing the widely varying image quality across all cohorts.

As for equipment-related variabilities, since the only cohort with available information about the scanner manufacturer is the source cohort (NACC), we visualized model-generated embeddings of MRIs coming from its

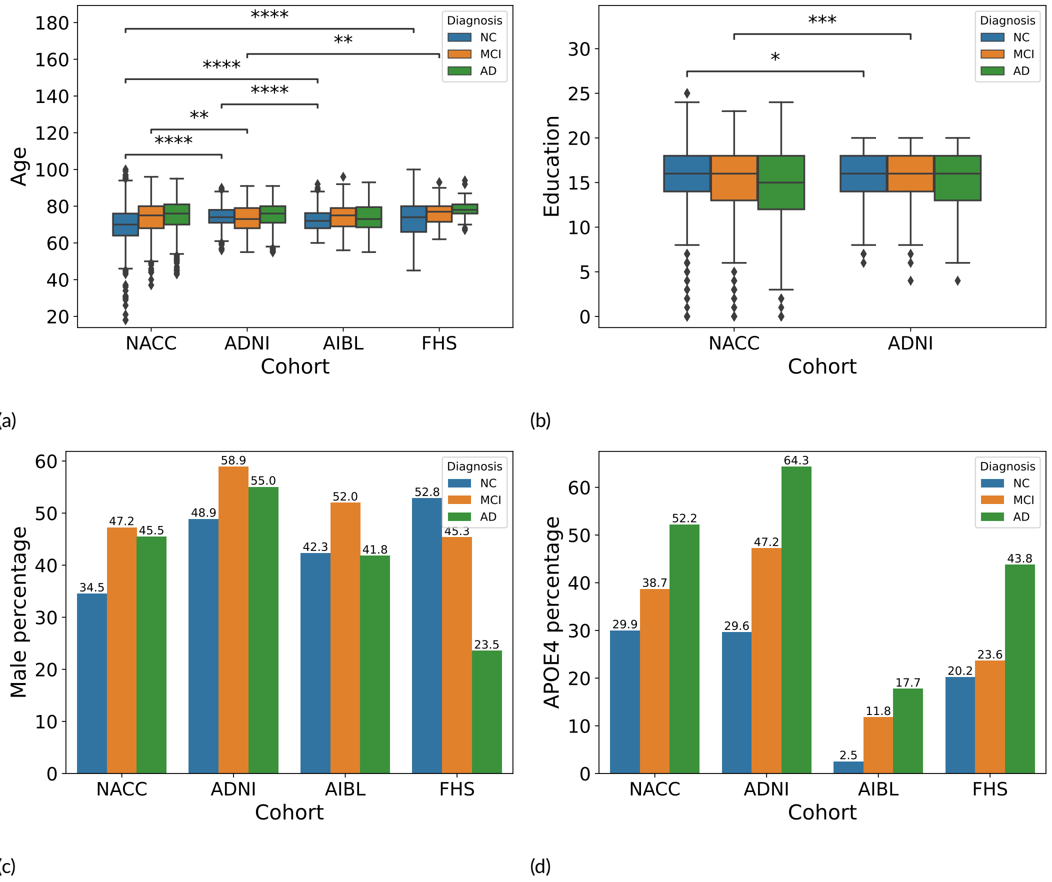

FIGURE S1 Distribution plots of patient demographics across the NACC ($n = 4,647$), ADNI ($n = 1,821$), AIBL ($n = 661$), and FHS ($n = 304$) cohorts. (a),(b),(c) include statistical significance results with the following corrected p-value annotations: *: $0.01 < p <= 0.05$; **: $0.001 < p <= 0.01$; ***: 1e-4 $< p <= 0.001$; ****: $p <=$ 1e-4. (d),(e) show the percentage of males and patients with the ApoE4 gene, respectively, across the four cohorts. Education information on the AIBL dataset was not available and is categorical for FHS.

test split ($n = 930$) in latent space in Fig. S3. We color-coded the t-SNE plots by diagnosis label (NC, MCI, and AD) and marked them based on the scanner manufacturer. Scans with available scanner manufacturer information came from either GE, Siemens, or Philips. For both the baseline and our method, the plots do not show any clustering of data points according to the brand of scanner manufacturer. On the other hand, we can see clustering of data points with the same diagnosis labels on both plots.

In conclusion, the results yielded by our demographics analysis showed statistically variant distributions of patient demographics between the source and target cohorts. Furthermore, our IQA study illustrated remarkable variance in image quality across cohorts, possibly induced by intensity artifacts and imaging equipment variabilities. We posit that variance in demographics and data quality is present in clinical datasets and is a potential, if not main, contributor to the distributional shift between cohorts.

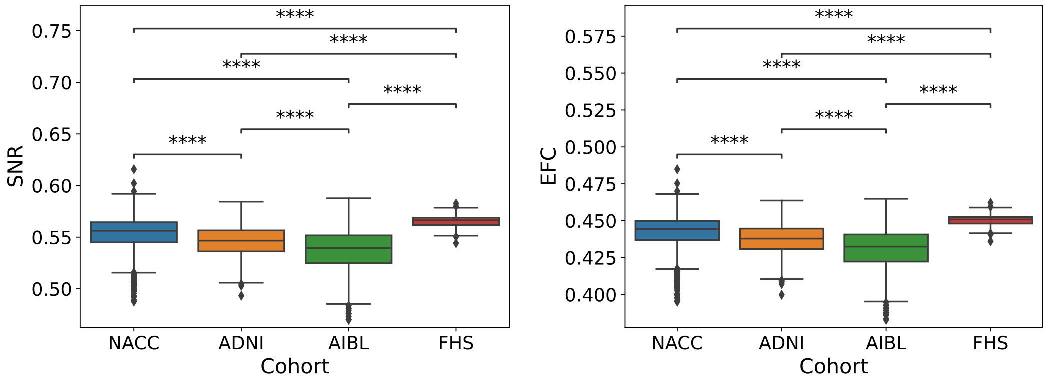

FIGURE S2 Image Quality Assesment (IQA) metrics of the preprocessed and normalized MRI scans over the NACC (n = 4,647), ADNI (n = 1,821), AIBL (n = 661), and FHS (n = 304) cohorts. Statisfical significance results of the Mann-Whitney U Test are displayed with the following annotations of corrected p-values: *: 0.01 < p <= 0.05; **: 0.001 < p <= 0.01; ***: 1e-3 < p <= 0.001; ****: p <= 1e-4.

B | RESULTS

To support our results in Table 2 of the main manuscript, we generated Receiver Operating Characteristic (ROC) and precision-recall (PR) curves based on model predictions on the target cohorts, ADNI, AIBL, and FHS. Fig. S8 shows the ROC curves and their corresponding area under the curve (AUC) values computed for our method (row 7 in Table 2) against the baseline[4] (row 1 in Table 2) and the other domain generalization (DG) methods, RSC[5] and Mixup[6]. The AUC values were computed for one versus rest as well as one versus one classification over five runs, with their standard deviation values. The results show that our model outperformed the baseline and state-of-the-art DG methods in the classification of all labels, with the exception of NC vs. rest on FHS and MCI vs. rest on ADNI, with a 0.3% and 1.1% difference in performance respectively. In cases where RSC and/or Mixup performed worse than or on par with the baseline, e.g., on the classification of MCI vs. rest and that of MCI vs. NC, our method consistly outperformed the baseline across the three cohorts.

Additionally, Fig. S9 shows the PR curves and their AUC values corresponding to the macro average precision score computed for each of the evaluated models across the ADNI, AIBL, and FHS cohorts. Our method showed a remarkably higher precision score than both the baseline and the DG methods for the classification of AD, reflecting the disease-informed nature of our computational framework.

We further explored the effect of demographic variance on model performance. To start, we sampled data from each of the four cohorts according to the different demographic variables we had access to. Fig. S4 depicts histogram plots of the categorical variables gender and ApoE4 positivity across the NACC test split (n = 930), ADNI (n = 930), AIBL (n = 661), and FHS (n = 304) cohorts. And Fig. S5 shows histogram plots of the continuous variables age and education across the same four cohorts. Plots in both figures were color-coded according to the diagnosis label.

We then evaluated performance of both the baseline (row 1 in Table 2 of the manuscript) and our model (row 7 in Table 2 of the manuscript) on the sub-samples matched for the demographic variables and displayed the results in Figs. S6 and S7. Model performance on sub-samples matched for gender and ApoE4 positivity is displayed in Fig. S6 and that on sub-samples matched for age and education in Fig. S7. It is important to note that each of the sub-samples have different distributions of the diagnosis labels NC, MCI, and AD. To this effect, we reported balanced accuracy, a more faith-

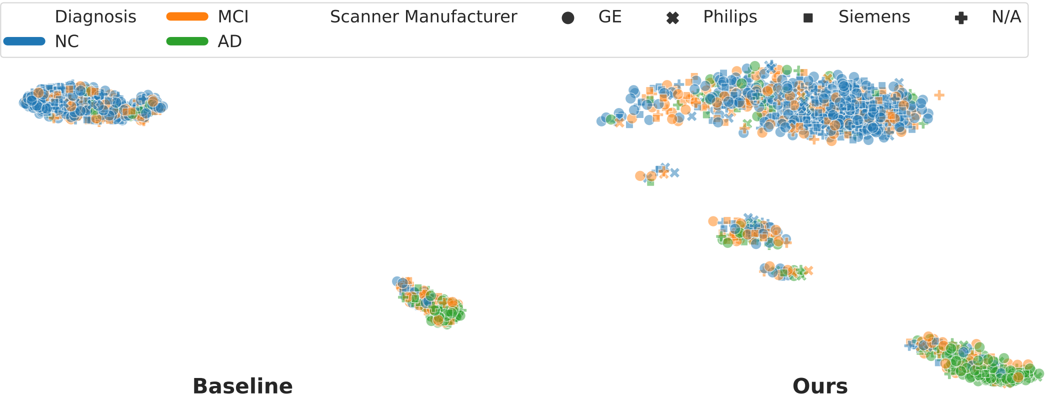

FIGURE S3 Visualization of the scanner manufacturer distribution across MRI embeddings from the NACC cohort. We generated MRI embeddings at the attention module level from two UNet3D models trained on the NACC cohort without domain generalization (**Baseline**, row 3 in Table 2 of the manuscript) and with our proposed DG framework (**Ours**, row 7 in Table 2 of the manuscript), and visualized them in a 2D space using t-SNE. For both models, data from the test split of the source cohort NACC (n = 930) were used. The data points were color-coded by diagnosis label and marked by scanner manufacturer. N/A indicates that the scanner manufacturer information was provided.

ful metric that takes class imbalance into account. As figures S6 and S7 depict, our model shows an overall decrease in the performance gap between the different sub-samples in each plot. In Fig. S6, the decrease is especially remarkable between sub-samples of the ApoE4 variable from the AIBL and FHS cohorts. The improvement in performance is also reflected by the line plots in Fig. S7 that show lesser variance between sub-samples for our model than for the baseline.

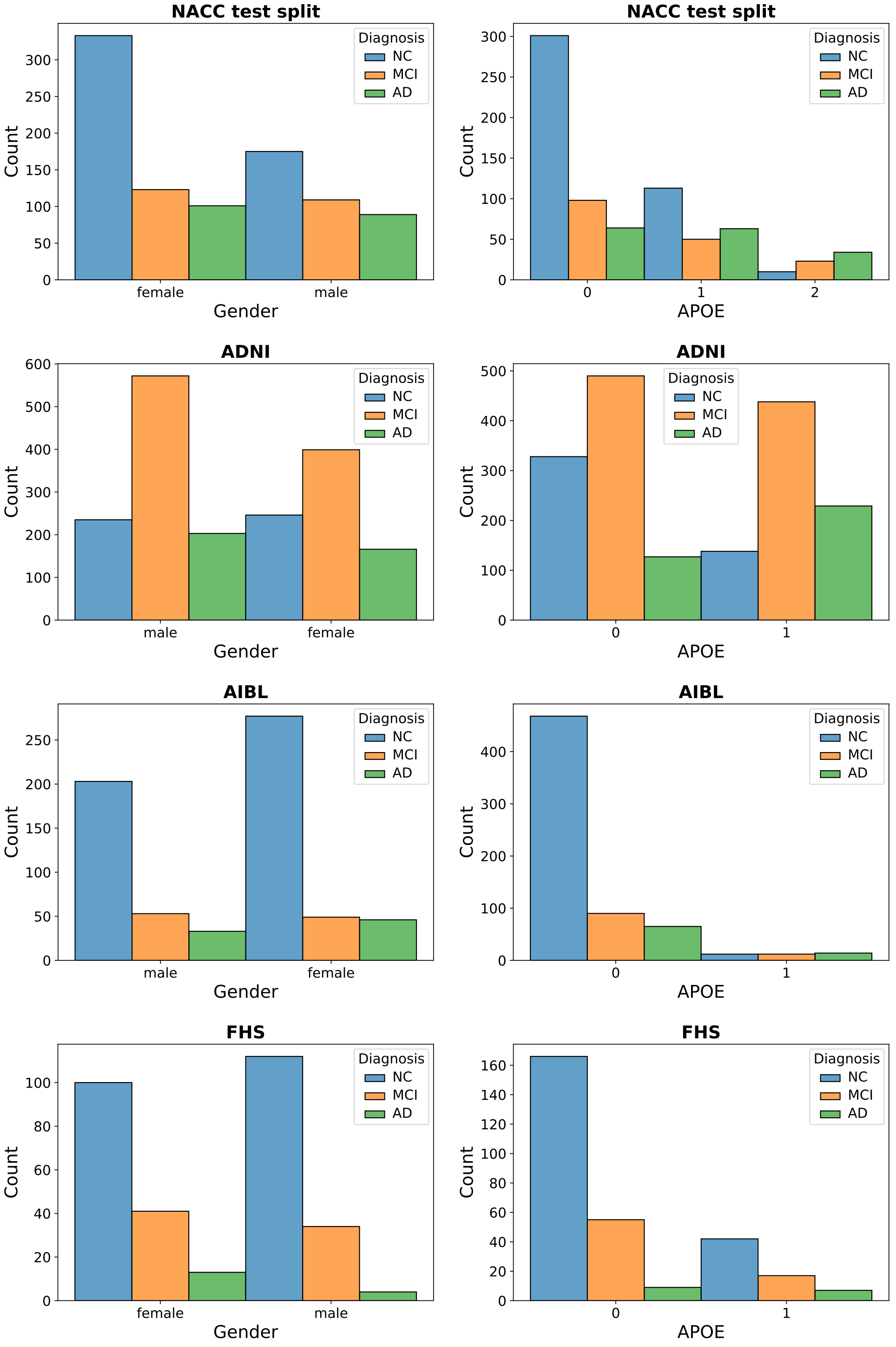

FIGURE S4 Histograms of the class distribution with respect to the categorical variables gender and APOE across the NACC test split (n = 930), ADNI (n = 1, 821), AIBL (n = 661), and FHS (n = 304) cohorts.

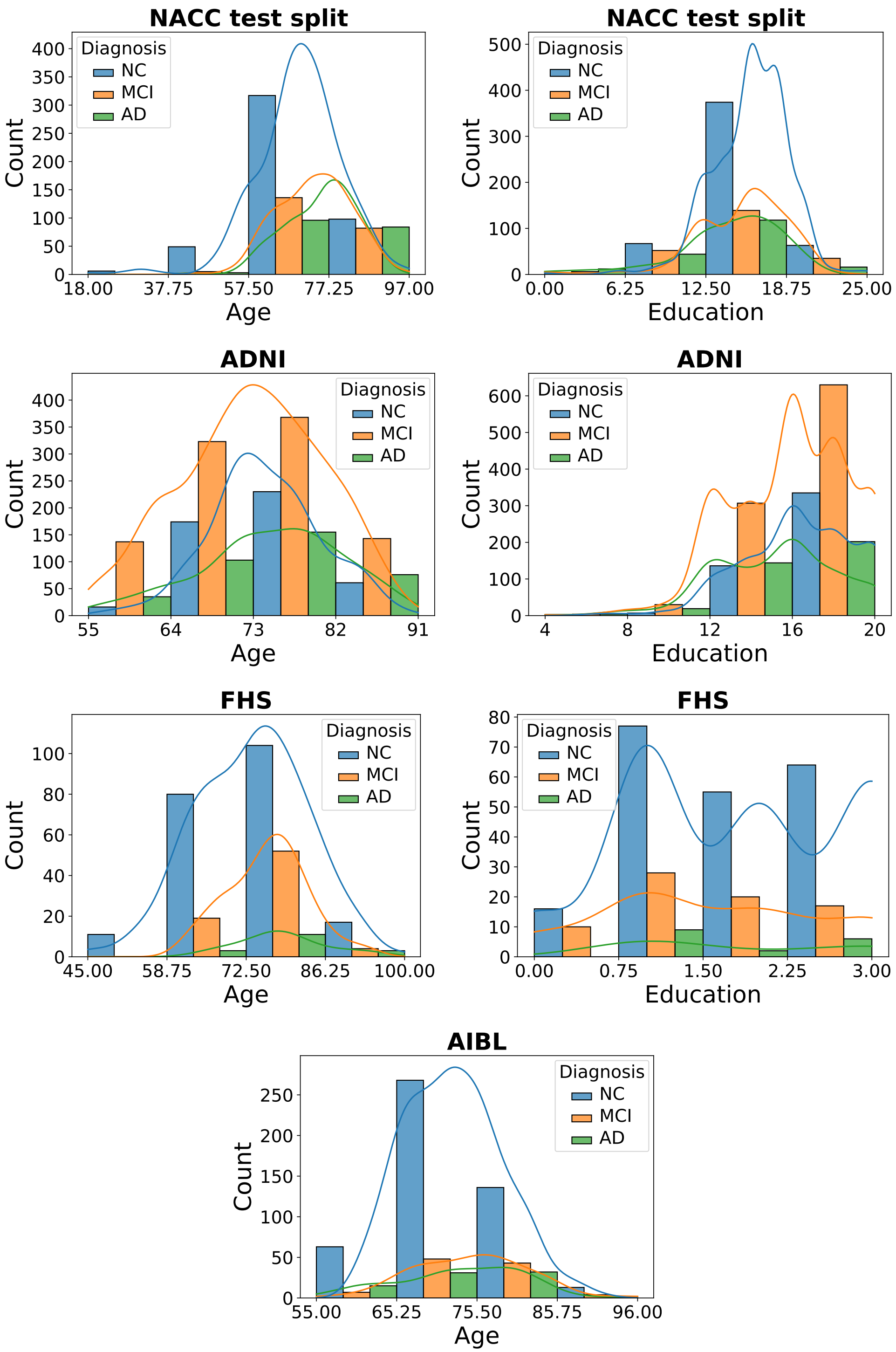

FIGURE S5 **Histograms of the class distribution over sub-samples matched with the continuous variables age and education across the NACC test split (n = 930), ADNI (n = 1,821), AIBL (n = 661), and FHS (n = 304) cohorts.** *Exception:* education information is not available for AIBL and is categorical for the FHS cohort, with the following categories: 0 = high school did not graduate, 1 = high school graduate, 2 = some college graduate, 3 = college graduate.

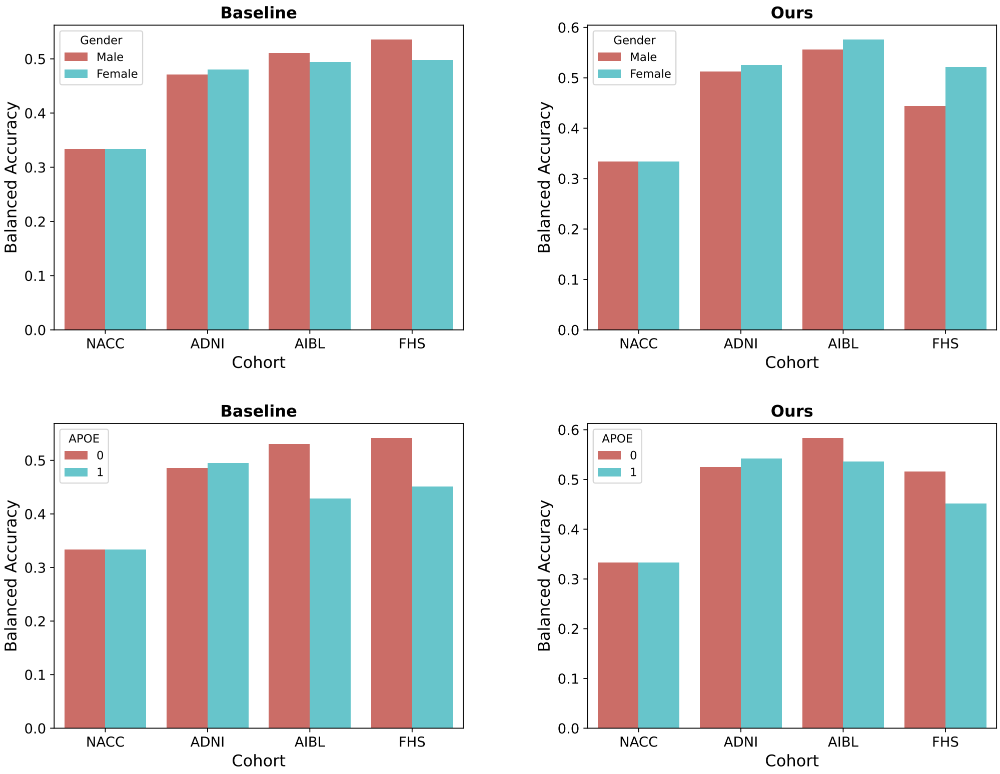

FIGURE S6 Model evaluation on sub-samples matched for the categorical variables gender and ApoE4 positivity on the ADNI ($n = 1,821$), AIBL ($n = 661$), and FHS ($n = 304$) cohorts. We display the balanced accuracies of the baseline (row 1 in Table 2) and our model (row 7 in Table 2).

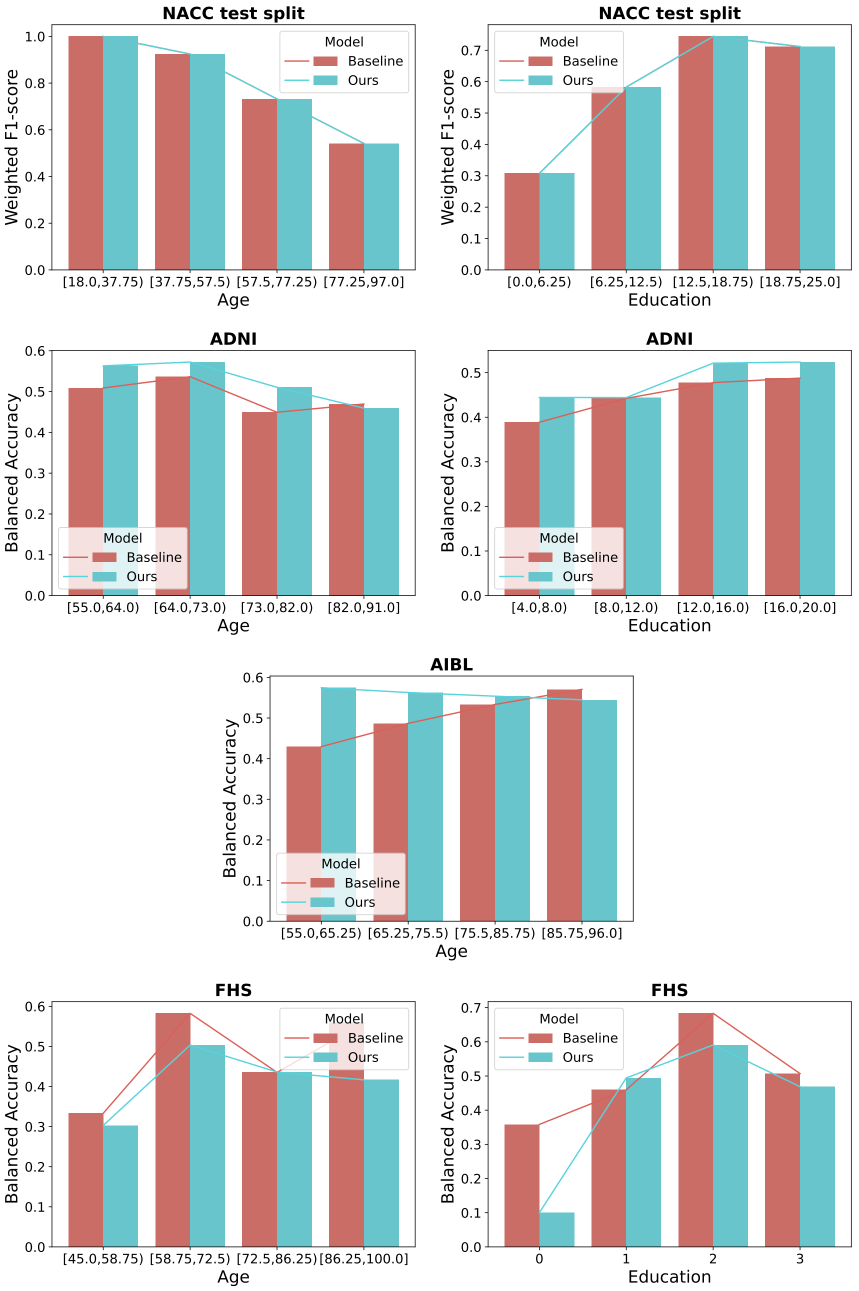

FIGURE S7 Model evaluation on sub-samples matched for age and education on the ADNI ($n = 1,821$), AIBL ($n = 661$), and FHS ($n = 304$) cohorts. We display the balanced accuracies of the baseline (row 1 in Table 2) and our model (row 7 in Table 2).

FIGURE S8 Receiver Operating Characteristic (ROC) curves calculated on the target cohorts, ADNI($n = 1,821$), AIBL ($n = 661$), and FHS ($n = 304$). We plotted the sensitivity, the true positive rate, versus the false negative rate (1-specificity) for predictions by models trained without DG [4], (row 3 of Table 2), RSC [5], Mixup [6], and our proposed DG framework (row 7 of Table 2). The rates were computed for one vs. rest and one vs. one classifications of each of the diagnosis labels, NC, MCI, and AD. The area under the curve denotes the mean accuracy and standard deviation computed over five runs. The best performing model is highlighted in bold in each of the SS curves.

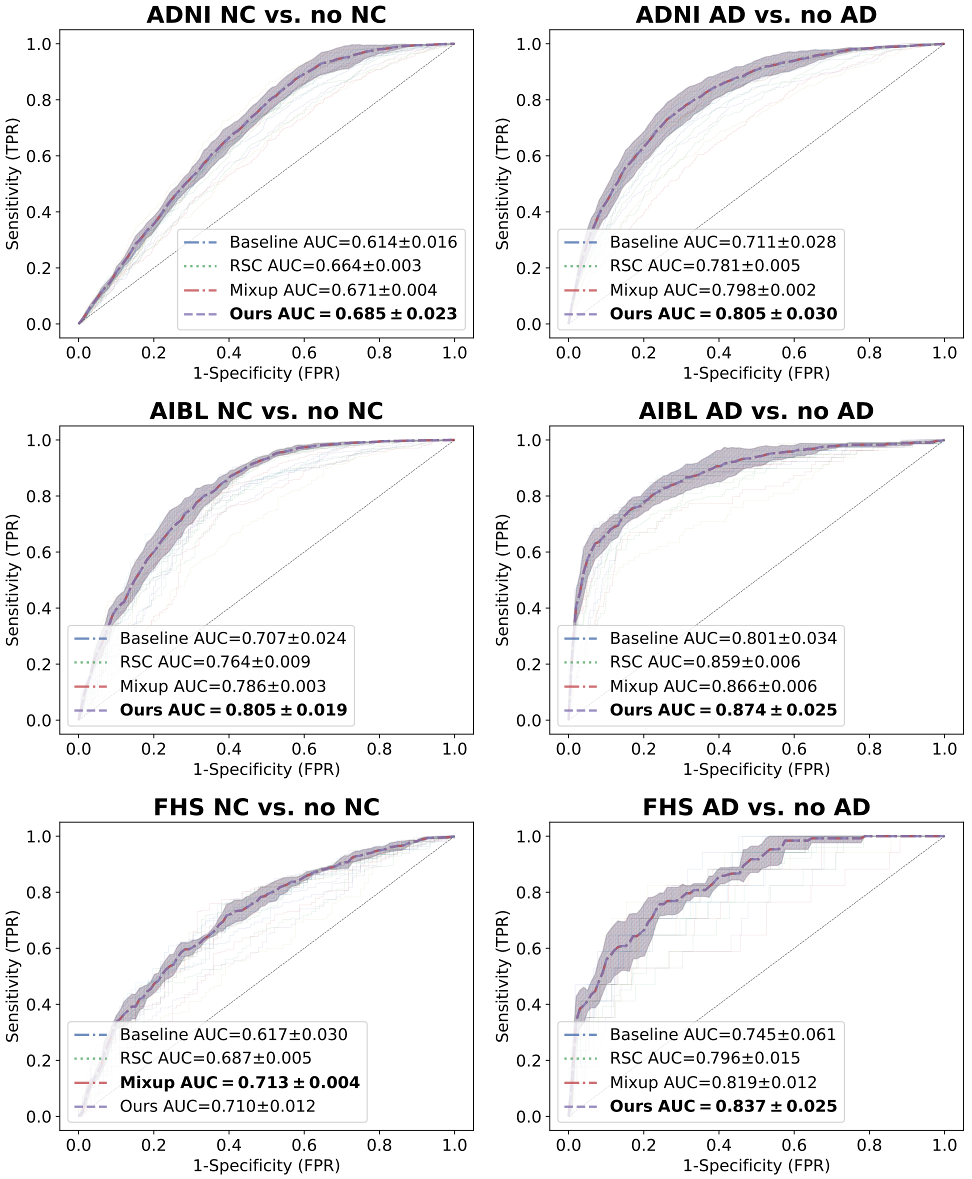
ADNI NC vs. no NC
ADNI AD vs. no AD
AIBL NC vs. no NC
AIBL AD vs. no AD
FHS NC vs. no NC
FHS AD vs. no AD
Sensitivity (TPR)
1-Specificity (FPR)
Baseline AUC=0.614±0.016
RSC AUC=0.664±0.003
Mixup AUC=0.671±0.004
Ours AUC = 0.685 ± 0.023
Baseline AUC=0.711±0.028
RSC AUC=0.781±0.005
Mixup AUC=0.798±0.002
Ours AUC = 0.805 ± 0.030
Baseline AUC=0.707±0.024
RSC AUC=0.764±0.009
Mixup AUC=0.786±0.003
Ours AUC = 0.805 ± 0.019
Baseline AUC=0.801±0.034
RSC AUC=0.859±0.006
Mixup AUC=0.866±0.006
Ours AUC = 0.874 ± 0.025
Baseline AUC=0.617±0.030
RSC AUC=0.687±0.005
Mixup AUC = 0.713 ± 0.004
Ours AUC=0.710±0.012
Baseline AUC=0.745±0.061
RSC AUC=0.796±0.015
Mixup AUC=0.819±0.012
Ours AUC = 0.837 ± 0.025

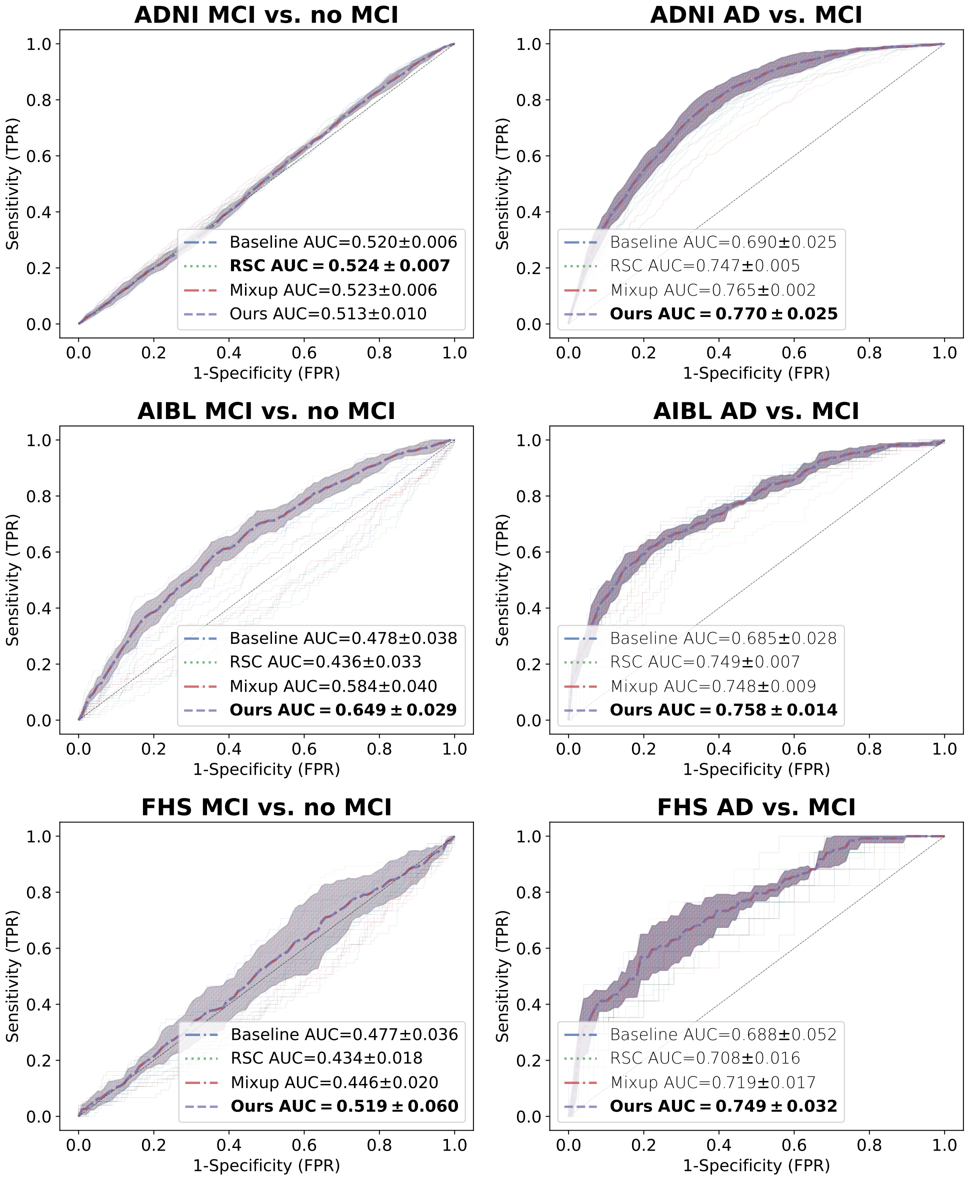
ADNI MCI vs. no MCI
Baseline AUC=0.520±0.006
RSC AUC = 0.524 ± 0.007
Mixup AUC=0.523±0.006
Ours AUC=0.513±0.010
ADNI AD vs. MCI
Baseline AUC=0.690±0.025
RSC AUC=0.747±0.005
Mixup AUC=0.765±0.002
Ours AUC = 0.770 ± 0.025
AIBL MCI vs. no MCI
Baseline AUC=0.478±0.038
RSC AUC=0.436±0.033
Mixup AUC=0.584±0.040
Ours AUC = 0.649 ± 0.029
AIBL AD vs. MCI
Baseline AUC=0.685±0.028
RSC AUC=0.749±0.007
Mixup AUC=0.748±0.009
Ours AUC = 0.758 ± 0.014
FHS MCI vs. no MCI
Baseline AUC=0.477±0.036
RSC AUC=0.434±0.018
Mixup AUC=0.446±0.020
Ours AUC = 0.519 ± 0.060
FHS AD vs. MCI
Baseline AUC=0.688±0.052
RSC AUC=0.708±0.016
Mixup AUC=0.719±0.017
Ours AUC = 0.749 ± 0.032
Sensitivity (TPR)
1-Specificity (FPR)

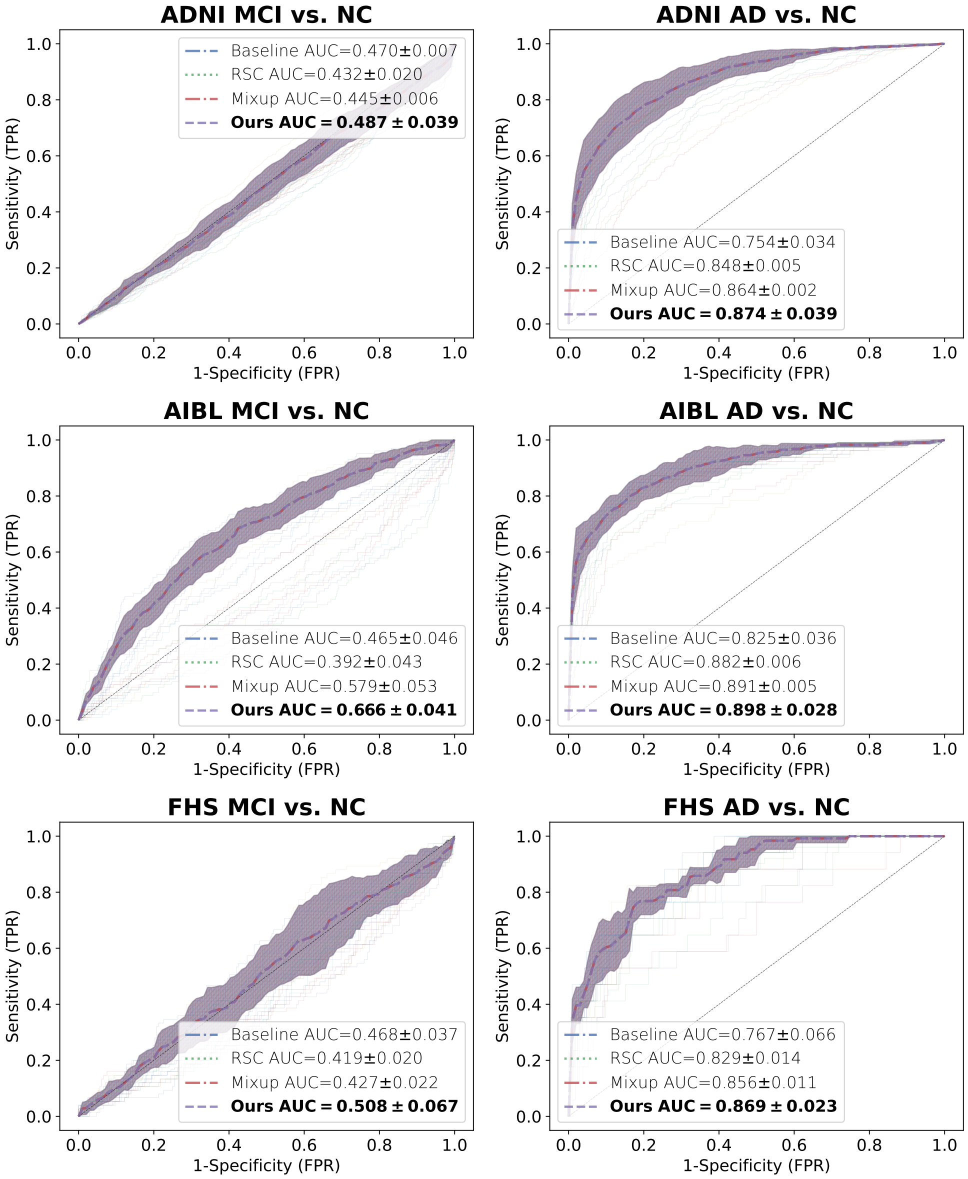
ADNI MCI vs. NC
Baseline AUC=0.470±0.007
RSC AUC=0.432±0.020
Mixup AUC=0.445±0.006
Ours AUC = 0.487 ± 0.039
ADNI AD vs. NC
Baseline AUC=0.754±0.034
RSC AUC=0.848±0.005
Mixup AUC=0.864±0.002
Ours AUC = 0.874 ± 0.039
AIBL MCI vs. NC
Baseline AUC=0.465±0.046
RSC AUC=0.392±0.043
Mixup AUC=0.579±0.053
Ours AUC = 0.666 ± 0.041
AIBL AD vs. NC
Baseline AUC=0.825±0.036
RSC AUC=0.882±0.006
Mixup AUC=0.891±0.005
Ours AUC = 0.898 ± 0.028
FHS MCI vs. NC
Baseline AUC=0.468±0.037
RSC AUC=0.419±0.020
Mixup AUC=0.427±0.022
Ours AUC = 0.508 ± 0.067
FHS AD vs. NC
Baseline AUC=0.767±0.066
RSC AUC=0.829±0.014
Mixup AUC=0.856±0.011
Ours AUC = 0.869 ± 0.023
Sensitivity (TPR)
1-Specificity (FPR)

FIGURE S9 Precision-recall curves showing model performance on the target cohorts, ADNI ($n = 1,821$), AIBL ($n = 661$), and FHS ($n = 304$). We plotted the precision versus recall, negative rate (1-specificity) for predictions by models trained without DG [4](row 3 of Table 2), RSC[5], Mixup[6], and our proposed DG framework (row 7 of Table 2). The area under the curve denotes the macro average precision score and standard deviation computed over five runs.

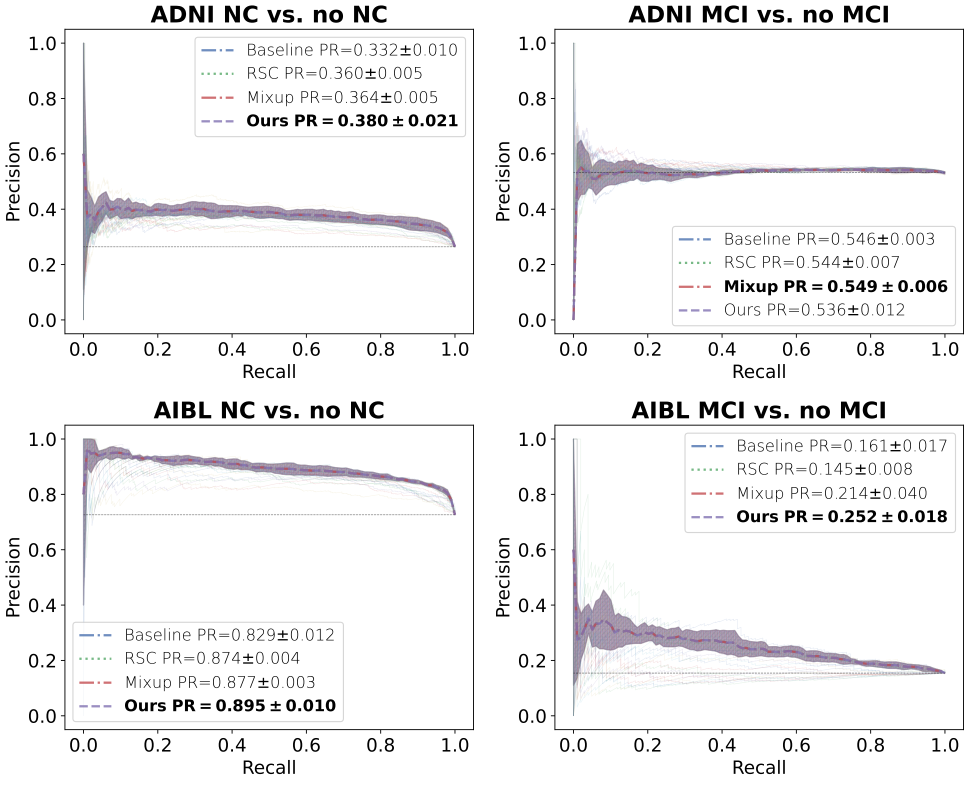
ADNI NC vs. no NC
Baseline PR=0.332±0.010
RSC PR=0.360±0.005
Mixup PR=0.364±0.005
Ours PR = 0.380 ± 0.021
Precision
Recall
ADNI MCI vs. no MCI
Baseline PR=0.546±0.003
RSC PR=0.544±0.007
Mixup PR = 0.549 ± 0.006
Ours PR=0.536±0.012
AIBL NC vs. no NC
Baseline PR=0.829±0.012
RSC PR=0.874±0.004
Mixup PR=0.877±0.003
Ours PR = 0.895 ± 0.010
AIBL MCI vs. no MCI
Baseline PR=0.161±0.017
RSC PR=0.145±0.008
Mixup PR=0.214±0.040
Ours PR = 0.252 ± 0.018

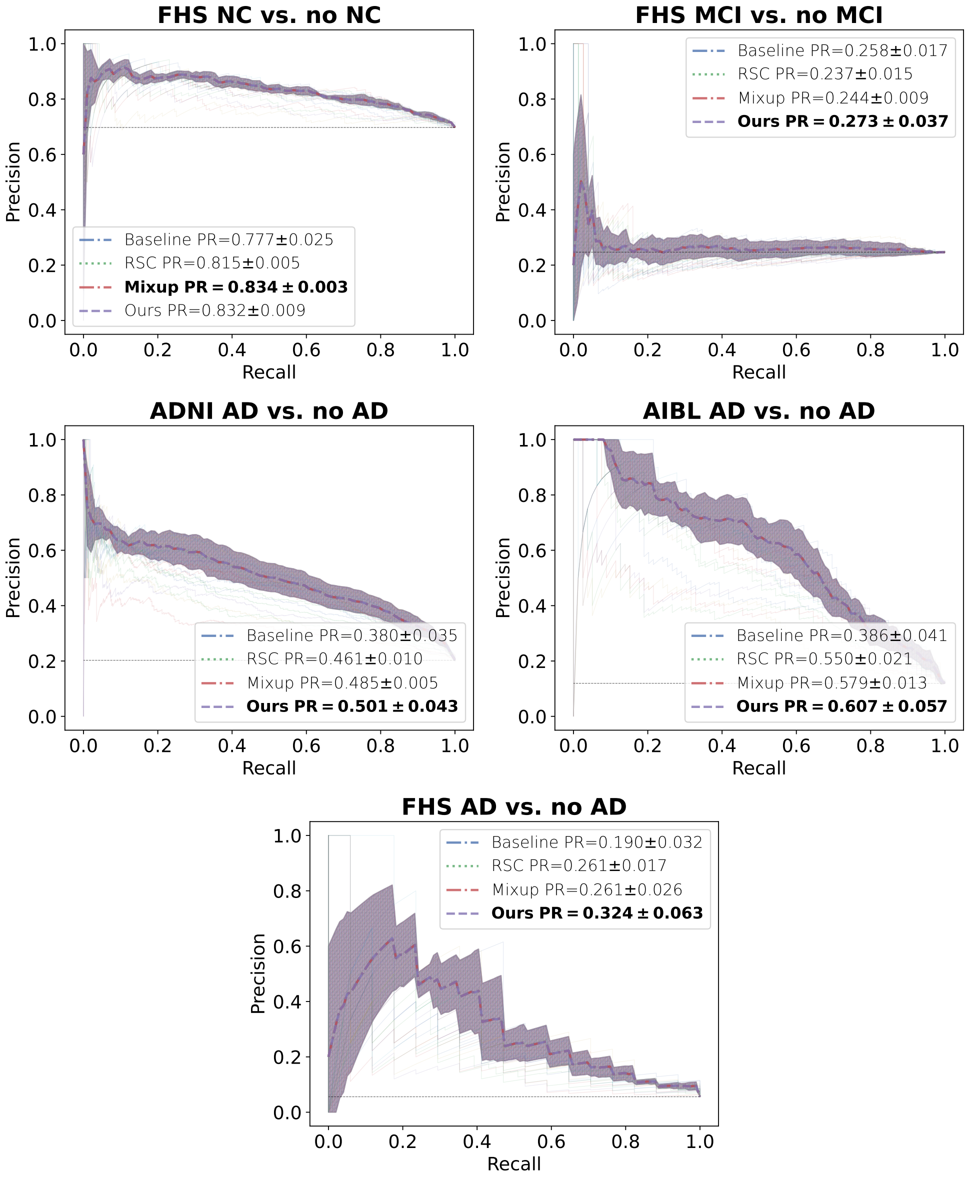
FHS NC vs. no NC
Baseline PR=0.777±0.025
RSC PR=0.815±0.005
Mixup PR = 0.834 ± 0.003
Ours PR=0.832±0.009
FHS MCI vs. no MCI
Baseline PR=0.258±0.017
RSC PR=0.237±0.015
Mixup PR=0.244±0.009
Ours PR = 0.273 ± 0.037
ADNI AD vs. no AD
Baseline PR=0.380±0.035
RSC PR=0.461±0.010
Mixup PR=0.485±0.005
Ours PR = 0.501 ± 0.043
AIBL AD vs. no AD
Baseline PR=0.386±0.041
RSC PR=0.550±0.021
Mixup PR=0.579±0.013
Ours PR = 0.607 ± 0.057
FHS AD vs. no AD
Baseline PR=0.190±0.032
RSC PR=0.261±0.017
Mixup PR=0.261±0.026
Ours PR = 0.324 ± 0.063
Precision
Recall

References

[1] Qiu S, et al. Multimodal deep learning for Alzheimer's disease dementia assessment. Nature communications 2022;13(1):3404.

[2] Magnotta VA, Friedman L, BIRN F. Measurement of signal-to-noise and contrast-to-noise in the fBIRN multicenter imaging study. Journal of digital imaging 2006;19:140–147.

[3] Atkinson D, Hill DL, Stoyle PN, Summers PE, Keevil SF. Automatic correction of motion artifacts in magnetic resonance images using an entropy focus criterion. IEEE Transactions on Medical imaging 1997;16(6):903–910.

[4] Zhou Z, et al. Models genesis: Generic autodidactic models for 3d medical image analysis. In: Medical Image Computing and Computer Assisted Intervention–MICCAI 2019: 22nd International Conference, Shenzhen, China, October 13–17, 2019, Proceedings, Part IV 22 Springer; 2019. p. 384–393.

[5] Huang Z, Wang H, Xing EP, Huang D. Self-challenging improves cross-domain generalization. In: Computer Vision–ECCV 2020: 16th European Conference, Glasgow, UK, August 23–28, 2020, Proceedings, Part II 16 Springer; 2020. p. 124–140.

[6] Zhang H, Cisse M, Dauphin YN, Lopez-Paz D. mixup: Beyond empirical risk minimization. arXiv preprint arXiv:171009412 2018;.
